# Sheep Pox Susceptibility: Role of Genetic Variants, Gene Expression, and Immune-Oxidative Markers

**DOI:** 10.3390/vetsci12090867

**Published:** 2025-09-08

**Authors:** Asmaa A. Darwish, Huda A. Alqahtani, Amin Tahoun, Ahmed Ateya, Noha A. Helmy, Amani A. Hafez, Hanan M. Alharbi, Khairiah M. Alwutayd, Manal A. Babaker, Ammar AL-Farga, Eman A. Al-Shahari, Zakaria A. Salih, Mohammed Ali. Al-Duais, Ahmed El-Sayed

**Affiliations:** 1 Department of Animal Health and Poultry, Animal and Poultry Production Division, Desert Research Center (DRC), Cairo 11753, Egypt; asmaa_vet25@drc.gov.eg (A.A.D.); amani.hafez@drc.gov.eg (A.A.H.); 2 Department of Zoology, College of Science, King Saud University, P.O. Box 2455, Riyadh 11451, Saudi Arabia; hudalqahtani@ksu.edu.sa; 3 Department of Animal Medicine, Faculty of Veterinary Medicine, Kafrelshkh University, Kafrelsheikh 33516, Egypt; amin.abdelhady@vet.kfs.edu.eg; 4 Department of Clinical Veterinary Medical Sciences, Jordan University of Science and Technology, Irbid 22110, Jordan; 5 Department of Development of Animal Wealth, Faculty of Veterinary Medicine, Mansoura University, Mansoura 35516, Egypt; 6 Veterinary Serum and Vaccine Research Institute, Abbasia, Cairo 11566, Egypt; nahelmy@arc.gov.eg; 7 Department of Biology, College of Science, Princess Nourah bint Abdulrahman University, P.O. Box 84428, Riyadh 11671, Saudi Arabia; hmalharbi@pnu.edu.sa (H.M.A.); kmalwateed@pnu.edu.sa (K.M.A.); 8 Department of Chemistry, Faculty of Science, Majmaah University, Al Majmaah 11952, Saudi Arabia; m.babaker@mu.edu.sa; 9 Department of Biological Sciences, College of Science, University of Jeddah, Jeddah 22233, Saudi Arabia; amalfarga@uj.edu.sa; 10 Health Specialties, Basic Sciences and Their Applications Unit, Applied College at Muhayil Asir, King Khalid University, Abha 61421, Saudi Arabia; ralshehaeri@kku.edu.sa; 11 Research and Training Station, King Faisal University, P.O. Box 400, Al-Ahsa 31982, Saudi Arabia; zahmed@kfu.edu.sa; 12 Department of Food Engineering and Technology, Faculty of Engineering and Technology, University of Gezira, Wad-Medani 5118 40466, Sudan; 13 Department of Biochemistry, Faculty of Science, University of Tabuk, Tabuk 71491, Saudi Arabia

**Keywords:** sheep pox, immunity, gene expression, antioxidants, SNPs

## Abstract

Sheep pox, caused by sheep pox virus (SPV), is a transboundary disease that threatens sheep production and trade. This study aimed to identify genetic, immunological, and biochemical markers associated with susceptibility to SPV in Barki ewes. A total of 100 adult ewes were examined, including 50 clinically healthy and 50 naturally infected animals. PCR detected SPV DNA in 60% of suspected scab samples, highlighting diagnostic challenges in field investigations. Blood samples were analyzed for hematological indices, cytokine profiles, acute phase proteins, oxidative stress biomarkers, iron metabolism, and hormonal parameters. Expression profiles and single-nucleotide polymorphisms (SNPs) in 15 immune and antioxidant genes were characterized from cDNA-derived sequences. Infected animals exhibited microcytic hypochromic anemia, leukocytosis, elevated proinflammatory cytokines, and reduced IL-10. Acute phase proteins, oxidative stress markers, and cortisol were increased, whereas antioxidant capacity and transferrin were reduced. Twenty-three SNPs were identified, including non-synonymous variants, which showed promising but unvalidated associations with disease status. These findings highlight immune, oxidative, and genetic alterations in SPV-infected sheep, but further longitudinal and cross-validated studies are needed to establish their diagnostic or breeding utility.

## Simple Summary

Sheep pox is a highly contagious viral disease of major economic concern in small ruminant production. This study investigated the genetic, transcriptional, and biochemical factors associated with susceptibility to sheep pox in Barki ewes. Blood and scab samples were analyzed for hematological changes, inflammatory and antioxidant markers, and single-nucleotide polymorphisms (SNPs) in key immune and antioxidant genes. PCR detected sheep pox virus DNA in 60% of suspected scab samples, indicating some diagnostic uncertainty under field conditions. Infected animals exhibited anemia, leukocytosis, elevated proinflammatory cytokines, oxidative stress, and altered iron metabolism. Twenty-three novel SNPs were detected in expressed regions of immune and antioxidant genes, some of which showed potential as markers of disease susceptibility. These findings provide preliminary insights into disease monitoring and selective breeding strategies, though further validation in larger and cross-validated datasets is required.

## 1. Introduction

The Barki sheep breed, named after the Libyan province Barka, is native to Egypt’s northwestern coastal zone and plays a vital role in supporting local communities in this region [1]. Its distribution covers a wide area extending from eastern Libya to the western parts of Alexandria, Egypt. This breed is well known for its strong adaptability to harsh environmental conditions, including limited forage availability, heat stress, and challenging climate factors [2].

Sheep pox virus (SPV), a member of the genus Capripoxvirus (family Poxviridae), is endemic across large parts of Africa, the Middle East, and Asia, where it continues to cause significant constraints on small ruminant production and trade. In Egypt, outbreaks are reported regularly, with morbidity rates ranging from 20% to over 60% depending on season, flock management, and vaccination status. The northwestern coastal zone, home to the indigenous Barki breed, represents one of the regions most affected by recurrent SPV incursions. Barki sheep are highly valued for their adaptability to arid and semi-arid conditions; however, they are also vulnerable to infectious diseases such as sheep pox, which threatens flock productivity, skin and wool quality, and farmer livelihoods. Recent molecular studies from Egypt and neighboring North African countries have confirmed the circulation of diverse SPV strains with close phylogenetic relationships, underscoring the persistent endemicity of the virus in this region. Despite vaccination campaigns, incomplete coverage, variable vaccine efficacy, and informal animal movement contribute to the continued spread of SPV among Barki flocks in Egypt’s coastal areas [3,4].

Clinically, the disease appears as papules that develop into nodules, vesicles, pustules, and eventually scabs, mainly located at the mucocutaneous junctions of the lips and incisor teeth, sometimes spreading to the buccal mucosa [5]. Occasionally, lesions can be found on the feet, vulva, perineum, and coronet. The severity and fatality of the disease vary depending on factors such as breed, immune status, viral strain, and viral load. In fully susceptible flocks, mortality rates can reach 50%, and young lambs may experience up to 100% mortality [4].

Transmission primarily occurs through inhalation or skin abrasions during contact between infected and susceptible animals [6]. Aerosols generated by coughing, sneezing, head shaking, vocalizations, and breathing facilitate spread [7]. Additionally, insect vectors like *Glossina* spp. and stable flies (*Stomoxys calcitrans*) can mechanically transmit the virus [8]. Indirect transmission may occur through contaminated feed, water, wool, or environments polluted by saliva, ocular and nasal discharges, skin lesions, scabs, urine, or feces from infected animals [9].

Susceptible sheep may also become infected through exposure to untreated skins, hides, or wool from diseased animals, which can harbor viable virus particles [10]. No specific antiviral treatment is currently available for sheep pox; thus, managing secondary bacterial infections through hygiene, adequate ventilation, and nutrition is essential [11]. Vaccination remains the primary method for preventing the disease [12].

Viral infections induce the production of reactive oxygen and nitrogen species as part of the immune response and cytokine activity [13]. Many pathogenic viruses act as pro-oxidants by increasing free radical generation, which contributes to their pathogenicity [14]. Oxidative stress from poxvirus infection damages cellular membranes and disrupts normal cell function [15]. This has led to interest in antioxidant therapies as potential treatments, underscoring the link between viral pathogenesis and oxidative damage [16].

Advances in molecular genetics have improved disease control by identifying genetic factors related to host resistance [17]. Numerous genetic markers, particularly single-nucleotide polymorphisms (SNPs), have been associated with susceptibility or resistance to infectious diseases in livestock [18,19,20]. These findings indicate that genetic differences among individuals influence their vulnerability to infection [21]. However, there is limited research on immunological and antioxidant changes, as well as genetic polymorphisms.

This study was designed as an exploratory, integrative assessment of clinical, biochemical, immunological, and genetic changes in sheep pox–infected animals. Rather than providing exhaustive methodological depth in any single domain, our goal was to generate a broad profile of host responses that can inform future focused investigations.

## 2. Materials and Methods

### 2.1. Animals and Study Design

This investigation involved 100 adult Barki ewes, aged between 3 and 8 years (mean ± SD: 5.1 ± 1.7), with body weights ranging from 27.5 to 45 kg (mean ± SD: 33.7 ± 5.8). The study was conducted during 2024 and 2025 with funding support from DRC-05-2-24 on privately owned farms situated along Egypt’s northwestern coastal region. The animals were classified into two groups: 50 clinically healthy ewes and 50 exhibiting symptoms of sheep pox infection.

Each animal underwent a full clinical assessment, including measurements of rectal temperature, pulse, and respiratory rate, following the protocol outlined by [22]. The control group (CG) was selected based on the presence of normal vital signs, absence of skin lesions, normal appetite and body weight, and no visible nasal or ocular discharge. On the other hand, the diseased group (DG) showed clinical signs such as fever, elevated heart and respiratory rates, reduced appetite and weight loss, along with rhinitis, conjunctivitis, and pronounced enlargement of superficial lymph nodes, notably the prescapular ones.

Some affected ewes developed noisy, labored breathing due to swollen retropharyngeal lymph nodes that compressed the upper airway. Additional symptoms included diarrhea and pulmonary complications. The hallmark skin lesions of sheep pox began as small, sharply defined hyperemic macules without fluid accumulation and with central depressions. These were mostly observed in hairless areas such as the lips, gums, forehead, eyelids, groin, axilla, udder, and scrotum. In more vulnerable animals, lesions extended across the body. These macules progressed into firm papules (0.5–1 cm in diameter). Mucosal papules often ulcerated, producing mucopurulent exudates from the mouth, anus, vagina, or prepuce. Most lesions became necrotic, leading to scab formation within 5–10 days. These scabs could remain for several weeks, eventually healing and leaving behind permanent scars.

The animals were housed in semi-covered shelters and received a daily ration consisting of concentrate feed and alfalfa hay, supplemented with seasonal green fodder when available. The diet was balanced to meet the maintenance and production requirements of adult Barki ewes, providing approximately 12–13% crude protein and adequate metabolizable energy, with unrestricted access to clean water.

Animals were initially classified as clinically diseased based on characteristic signs of sheep pox, including pyrexia, respiratory distress, lymphadenopathy, and the development of papular to nodular cutaneous lesions. Scab samples were collected for molecular confirmation using PCR. Of the 50 animals with clinical signs, 30 tested positive for SPV DNA. For the primary analyses, all clinically affected animals were included to reflect the field conditions under which diagnosis is usually made. However, to address potential misclassification bias, a sub-analysis restricted to PCR-positive animals was also performed, and the results are presented separately.

Case definitions and study groups. For the primary analysis, a virological gold standard was applied. Animals were classified as SPV cases if they exhibited compatible clinical signs (fever, papular/nodular/ulcerative skin lesions, lymphadenopathy and/or respiratory signs) and tested PCR-positive for SPV DNA from scab/lesion material (n = 30). Controls were clinically healthy animals sampled contemporaneously on the same farms and confirmed PCR-negative for SPV (n = 50). Animals with clinical signs but PCR-negative results were excluded from the primary analysis but were included in a prespecified sensitivity analysis (see below).

Sensitivity analysis. To assess robustness to potential misclassification, we repeated all outcome comparisons after reclassifying clinically suspected/PCR-negative animals as diseased. Results from both the biochemical/immunological are presented in the Results and summarized in Supplementary Table S1 (clinical/biochemical/immunological endpoints).

Sampling frame. The analytical dataset used a planned 1:1 case: control sampling ratio at enrollment; this does not represent morbidity. Farm-level morbidity was estimated separately as the proportion of animals with compatible clinical signs among the at-risk population during the outbreak period (see Epidemiological Description). This estimate is now reported with the numerator, denominator, farm count, and outbreak dates.

### 2.2. Sampling

#### 2.2.1. Sample Selection and Exposure

A total of 100 Barki ewes were sampled from private farms experiencing active sheep-pox (SPV) outbreaks during 2024–2025. Both symptomatic (infected) and asymptomatic (uninfected) animals were co-housed within the same farms, ensuring natural exposure of all animals to the virus. Asymptomatic ewes were confirmed uninfected using PCR testing to rule out subclinical infection. Animals were selected based on their clinical status and confirmed SPV exposure, allowing comparison between infected and exposed-but-uninfected ewes

#### 2.2.2. Blood Samples

Jugular blood samples were collected from each ewe using sterile syringes into both plain tubes and tubes containing EDTA. The samples collected were immediately placed on ice and transported to the laboratory. Blood and tissue samples were processed as previously described by [23] with minor modifications. Briefly, blood samples were centrifuged to separate serum, and tissue homogenates were prepared for downstream molecular and biochemical analyses [23]. Hematological analyses were performed using an automated hematology analyzer (Sysmex XP-300, Sysmex Corporation, Kobe City, Japan), which was calibrated daily according to the manufacturer’s recommendations using species-specific quality control material. Biochemical parameters were measured with a fully automated biochemistry analyzer (Mindray BS-240, Mindray, Shenzhen, China), with calibration performed prior to each analytical batch using manufacturer-supplied standards and control sera. Reference intervals for hematological and biochemical values were based on established ranges for Barki sheep maintained under Egyptian conditions and were used to guide interpretation of deviations observed in diseased versus control groups.

#### 2.2.3. Scab Sample Collections and Processing

Fifty scab samples were randomly collected from sheep exhibiting clinical signs suggestive of sheep pox. Approximately 3 g of tissue were carefully excised from the area beneath the tail using sterile scalpels, ensuring aseptic technique. Each sample was immediately transferred into a sterile universal container filled with 50% phosphate-buffered saline A (PBSA), adjusted to a pH between 7.2 and 7.6. To minimize bacterial and fungal contamination, phosphate-buffered saline A (PBSA) was supplemented with penicillin-streptomycin (100 IU penicillin G, 100 µg/mL streptomycin, and 100 µg/mL dihydrostreptomycin sulfate) and nystatin (50 IU/mL). Samples were labeled with unique identification codes and transported in an icebox to the Desert Research Center for further processing. All field sampling and laboratory procedures involving SPV material were reviewed and approved by the Desert Research Center Institutional Animal Care and Use Committee/Institutional Biosafety Committee (Approval code: code DRC-05-2-24) and were conducted in compliance with BSL-II guidelines.

Upon arrival, samples were thawed at 25 °C and washed three times with sterile 50% PBSA supplemented with the same antibiotics under a biosafety level II (BSL-II) cabinet. From each sample, approximately 1 g of tissue was placed into a sterile Petri dish, then manually ground using a sterile mortar and pestle in the presence of 9 mL sterile PBSA. The resulting homogenate was transferred into a sterile vacutainer tube and centrifuged at 3500 rpm for 10 min at 4 °C. After centrifugation, approximately 1 mL of the supernatant was aliquoted into cryovials, sealed, covered with aluminum foil, labeled accordingly, and stored at −20 °C until used for molecular investigations [24,25].

### 2.3. Extraction of Genomic DNA and PCR Amplification

#### 2.3.1. Molecular Detection of SPV

DNA was extracted from scab material using the QIAamp DNA Mini Kit (Qiagen, Hilden, Germany), including blank swab extraction controls with each batch. No-template controls were run in parallel with every PCR to detect potential contamination. PCR targeting the ORF103 gene (capsid protein) was performed according to manufacturer instruction. To enhance diagnostic sensitivity, a second independent assay targeting the RPO30 gene was conducted on a subset of samples (n = 100); positivity was defined as amplification in either assay. Amplicons were visualized by agarose gel electrophoresis and confirmed by sequencing (hyperlinks included in Data Availability Section). Primer Design All primers were synthesized by Metabion, Planegg, Germany and are listed in Table 1.

#### 2.3.2. PCR Conditions

The PCR reactions were carried out in a total volume of 25 µL, comprising 12.5 µL of EmeraldAmp Max PCR Master Mix (Takarazuka, Japan), 1 µL of each primer (20 pmol), 5.5 µL of nuclease-free water, and 5 µL of extracted DNA. Thermal cycling was conducted using an Applied Biosystems 2720 thermal cycler.

#### 2.3.3. Gel Electrophoresis of PCR Products

Amplified DNA fragments were separated on a 1.5% agarose gel (Applichem, Darmstadt, Germany) using 1× TBE buffer at ambient temperature and a voltage of 5 V/cm. Each gel well was loaded with 15 µL of PCR product. A 100 bp DNA ladder (GeneRuler, Thermo Fisher, Rheinfelden, Germany) served as a molecular size marker. Gels were visualized using a gel documentation system (Alpha Innotech, Biometra, San Leandro, CA, USA), and results were analyzed using compatible software.

### 2.4. Gene Sequencing and Phylogenetic Analyses

PCR products were purified using QIAquick PCR Product extraction kit. (Qiagen, Valencia, Madrid). Bigdye Terminator V3.1 cycle sequencing kit (Perkin-Elmer, Shelton, CT, USA) was used for the sequence reaction and then it was purified using Centrisep spin column. DNA sequences were obtained by Applied Biosystems3130 genetic analyzer (HITACHI, Ibaraki, Japan), a BLAST^®^ analysis (Basic Local Alignment Search Tool) [27] was initially performed to establish sequence identity to GenBank accessions. The sequence identities were determined by Lasergene DNAStar version 12.1 [28] and phylogenetic analyses were performed using maximum likelihood, neighbor joining and maximum parsimony in MEGA7 [29].

### 2.5. Total RNA Extraction, Reverse Transcription and Quantitative Real-Time PCR

Total RNA was extracted from sheep whole blood using TRIzol reagent, following the instructions provided with the RNeasy Mini Kit (Cat. No. 74104). For five minutes, 100 μL of fresh blood was vigorously mixed with 300 μL of TRI reagent. Before transferring the supernatant into an RNase-free tube, particle material was removed by centrifuging the mixture at 10,000–16,000× *g* for one minute. The lysed material was thoroughly mixed with an equivalent volume of ethanol (95–100%) in TRI reagent. After being moved into a Zymo-spin column in a collection tube, the mixture was centrifuged for 30 s at 10,000–16,000× *g*. Following centrifugation, the purification column was reinserted into the collection tube and the flow through was disposed of. Before centrifuging, 400 μL of Direct-zol RNA pre-wash was applied to the column. This process was repeated once the flow-through was discarded. To guarantee that the wash buffer was completely removed, 700 μL of RNA wash buffer was added to the column and centrifuged for two minutes. With caution, the column was moved into a tube devoid of RNase. A 50 μL volume of DNase/RNase-free water was introduced straight to the column matrix and centrifuged in order to elute RNA.

RNA concentration and purity were assessed using a NanoDrop^®^ ND-1000 spectrophotometer. First-strand complementary DNA (cDNA) was synthesized using the Revert Aid First Strand cDNA Synthesis Kit (Thermo Fisher, Catalogue No. EP0441 Waltham, MA, USA, Cat. No. EP0441) in accordance with the manufacturer’s instructions.

Quantitative real-time PCR (qPCR) was conducted to evaluate the relative expression of genes involved in immune response (IL-1α, IL-1β, IL-6, TNF-α, IFN-γ, IL-10, CHL1, CD46) and antioxidant defense (PRDX2, PRDX6, Nrf2, Keap1, OXSR1, SERP2, STIP1). Primers were designed based on *Ovis aries* sequences retrieved from the NCBI database and are listed in Table 2. The qPCR was performed using the SensiFAST™ SYBR Green PCR Master Mix (Bioline, Cat. No. BIO-98002) following the manufacturer’s instructions. Amplification was carried out in an Applied Biosystems StepOnePlus Real-Time PCR System (Thermo Fisher Scientific, Waltham, MA, USA) with 40 cycles under the following conditions: 95 °C for 15 s, annealing at primer-specific temperature for 30 s, and 72 °C for 30 s. β-actin was used as the housekeeping gene for normalization. Relative gene expression was calculated using the 2^−ΔΔCt^ method. PCR efficiency for all primers was confirmed to be between 90 and 110%, and melt-curve analysis verified the specificity of amplicons, each producing a single peak.

As a constitutive control, the housekeeping gene ß. actin was used for standardization. 3 µL of total RNA, 4 µl of 5× Trans Amp buffer, 0.25 µL of reverse transcriptase, 0.5 µL of each primer, 12.5 µL of 2× Quantitect SYBR green PCR master mix, and 8.25 µL of RNase-free water were all included in the 25 µL reaction mixture. The completed reaction mixture was put in a heat cycler, and the following procedure was carried out: 30 min at 50 °C for reverse transcription, 10 min at 94 °C for primary denaturation, 40 cycles at 94 °C for 15 s, 1 min at the annealing temperature as shown in Table 2, and 30 s at 72 °C. To confirm the PCR product’s specificity, a melting curve analysis was performed at the end of the amplification step. The 2^−ΔΔCt^ method was used to calculate each gene’s relative expression in each sample with respect to the ß. actin gene [30].

### 2.6. DNA Sequencing of Real-Time PCR Products (RT-PCR) and Polymorphism Detection

Primer dimers, nonspecific bands, and other contaminants were eliminated prior to DNA sequencing. Purification of real-time PCR products with predicted size (target bands) was performed using a PCR purification kit in accordance with the manufacturer’s instructions (Jena Bioscience # pp-201×s, Munich, Hamburg, Germany), as explained [31]. The Nanodrop (Uv-Vis spectrophotometer Q5000, Waltham, MA, USA) was used to quantify the PCR product in order to guarantee sufficient concentrations and purity of the PCR products as well as good product yield [32]. Using an ABI 3730XL DNA sequencer (Applied Biosystem, Waltham, MA USA) and the enzymatic chain terminator technique created by Sanger et al. [33]. PCR products with the target band were sent for forward and reverse DNA sequencing in order to find SNPs in the genes examined in control and pox affected ewes.

The software programs chromas 1.45 and blast 2.0 were used to analyze the DNA sequencing data [27]. Variations between the PCR results of the genes under investigation and the reference sequences found in GenBank were categorized as single-nucleotide polymorphisms (SNPs). Based on the alignment of DNA sequencing data, differences in the amino acid sequence of the genes being studied among the enrolled sheep were examined using the MEGA6 software program [34].

### 2.7. Biochemical, Antioxidant and Immunological Parameters

Serum biochemical parameters were measured using commercially available kits (MyBioSource company^®^, San Diago, CA, USA). Cytokines and acute phase proteins were quantified using ovine-specific or small ruminant–validated ELISA kits (MyBioSource company^®^, San Diago, CA, USA). Where manufacturer validation data were not available for sheep, intra- and inter-assay coefficients of variation were determined using pooled ovine serum samples, and values were confirmed against published reference ranges for sheep to ensure reliability. All concentrations are reported in SI units. Serum samples were analyzed in duplicate at recommended dilutions (typically 1:10 for cytokines and hormones, 1:50 for acute phase proteins, and 1:100 for ferritin, Cp, and Tf). The mean value of duplicates was used for statistical analysis. Runs were accepted only if the intra-assay coefficient of variation (CV) was <10% and the inter-assay CV < 15%.

### 2.8. Statistical Analysis

All statistical analyses were performed using SPSS software (version 23.0; IBM Corp., Armonk, NY, USA).

Primary and secondary outcomes. The study’s primary outcomes included hematological indices (RBC, Hb, PCV, TLC), key biochemical markers (glucose, AST, ALT, ALP, calcium, phosphorus, zinc, and copper), proinflammatory cytokines (IL-1β, IL-6, IFN-γ), acute phase proteins (haptoglobin, serum amyloid A), and selected SNPs in immune/antioxidant genes. Secondary outcomes included antioxidant capacity, oxidative stress indices, iron-binding parameters, and additional SNPs.

Data screening. Prior to statistical testing, all data were examined for missing values and outliers. Missing values represented <3% of the dataset and were excluded using listwise deletion. Outliers were identified using the interquartile range method (values beyond 1.5× IQR) and confirmed by visual inspection of boxplots and Q–Q plots. Outliers consistent with plausible biological variation were retained; those attributable to technical error were removed after checking raw records.

Assumption checks. Distributional assumptions were evaluated using the Shapiro–Wilk test (Supplementary Table S1). Homogeneity of variances was tested using Levene’s test. When assumptions were met, comparisons between groups were performed with independent-samples *t*-tests. When assumptions were violated, variables were log-transformed and re-tested; if assumptions remained unmet, non-parametric tests (Mann–Whitney U) or bias-corrected bootstrapping were applied.

Multiple comparisons. To account for multiple testing across numerous outcomes, the Benjamini–Hochberg false discovery rate (FDR) correction (q < 0.05) was applied. For transparency, Bonferroni-adjusted *p*-values are additionally reported in the Supplementary Tables.

Effect sizes and confidence intervals. Effect sizes were calculated for all comparisons: Cohen’s d (parametric tests) or rank-biserial correlation/Cliff’s delta (non-parametric tests). Each effect size is presented with its 95% confidence interval to facilitate interpretation of biological relevance.

Diagnostic accuracy. Receiver operating characteristic (ROC) curves were generated to evaluate diagnostic performance of selected biomarkers. Linear discriminant analysis (LDA) was repeated with 10-fold cross-validation and bootstrap resampling to reduce overfitting. Optimal cut-off values were determined using Youden’s index, and the area under the curve (AUC) with 95% confidence intervals was reported. Internal validation was performed with 10-fold cross-validation and bootstrap resampling (1000 replicates). A *p*-value < 0.05 was considered statistically significant unless otherwise adjusted for multiple comparisons.

## 3. Results

### 3.1. Pathological Evolution of Sheep Pox Lesions in Naturally Infected Sheep

Infected sheep typically developed papular lesions measuring between 0.5 and 1.0 cm in diameter, which in some cases progressed to nodules reaching 2–3 cm. Facial involvement was the most common presentation, observed in nearly 70% of the affected animals, followed by tail-base lesions in around 40%. Widespread cutaneous distribution, extending to multiple body regions, occurred in approximately 25% of severely affected cases.

**Figure 1:**
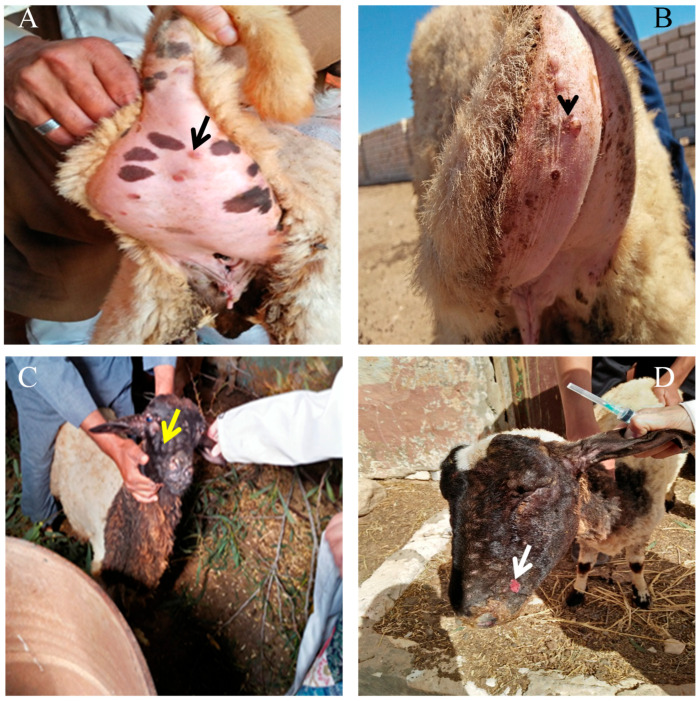
Progression of Sheep Pox Virus Lesions in Infected Sheep. (**A**) Initial lesion stage: Papular lesion localized at the fatty tail region (black arrow), indicating the primary site of viral infection. (**B**) advanced papular lesions in the tail (black arrowheads) (**C**) Early facial involvement as emerging papular lesion with mild edema (yellow arrow) (**D**) Ulcerative stage: Ulcerated nodular lesions (white arrows) with extensive skin damage and necrosis. Scale bars = 1 cm. Photographs reproduced with permission from animal owners.

The clinical progression of sheep pox virus (SPV) infection in naturally infected sheep exhibited distinct stages of cutaneous and systemic involvement. Initially, papular lesions developed in the fatty tail region, indicating the primary site of viral entry. As the infection progressed, lesions appeared on the facial skin, accompanied by mild edema. These gradually coalesced into nodular formations, covering much of the face (Figure 1). In the advanced stage, the nodular lesions ulcerated, resulting in extensive necrosis and tissue damage (Figure 1).

**Figure 2:**
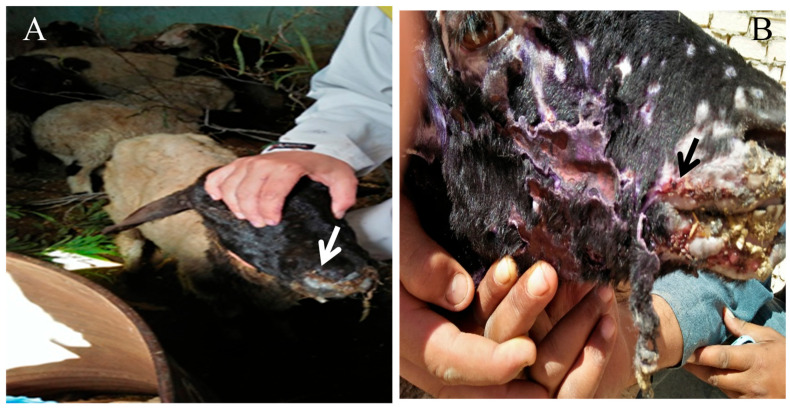
Systemic and progressive clinical signs of sheep pox virus infection in sheep. (**A**) Respiratory involvement: Severe respiratory distress characterized by nasal discharge (white arrow) and open-mouth breathing, suggesting viral lesions in the upper respiratory tract possibly complicated by secondary bacterial infection. (**B**) Severe suppurative lesions: Suppurative and necrotic lesions (black arrow) on the face and oral commissure, indicating bacterial complications superimposed on viral infection. Scale bars = 1 cm. Photographs reproduced with permission from animal owners.

Systemic manifestations were also observed. Infected sheep showed signs of severe respiratory distress, including open mouth breathing and nasal discharge ranging from serous to mucopurulent (Figure 2), suggesting upper respiratory tract involvement and secondary bacterial infections. Suppurative lesions were also noted around the mouth and facial region, supporting the presence of bacterial complications superimposed on the viral infection (Figure 2).

### 3.2. Genetic Variants and SNP Analysis

SNP analysis of candidate genes revealed several variants present in both infected and uninfected ewes. Some SNPs were observed more frequently in uninfected animals. However, these associations do not imply causality, as functional validation of these variants was not performed. The results indicate potential genetic associations with susceptibility to SPV, which warrant further investigation.

### 3.3. Hematological Parameters

**Figure 3:**
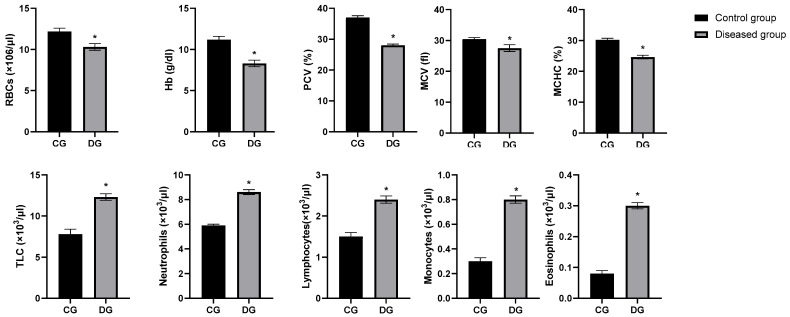
Comparative hematological indices of the control (CG) and diseased (DG) groups. Data are presented as mean ± SE for red blood cells (RBCs), hemoglobin (Hb), packed cell volume (PCV), mean corpuscular volume (MCV), mean corpuscular hemoglobin concentration (MCHC), total leukocyte count (TLC), and leukocyte differentials (neutrophils, lymphocytes, monocytes and eosinophils). Significant differences relative to the control group are marked with an asterisk (*p* < 0.05).

As presented in Figure 3, hematological assessment revealed significant deviations in the diseased group (DG) compared to the control group (CG). Red blood cell count (RBCs), hemoglobin concentration (Hb), and packed cell volume (PCV) were markedly reduced (*p* < 0.05), indicating the presence of anemia. Specifically, RBCs declined from 12.23 ± 0.45 to 10.34 ± 0.40 × 10^6^/μL, Hb from 11.25 ± 0.78 to 8.38 ± 0.46 g/L, and PCV from 37.18 ± 1.21 to 28.24 ± 0.40%. Mean corpuscular volume (MCV) and corpuscular hemoglobin concentration (MCHC) also decreased significantly, whereas mean corpuscular hemoglobin (MCH) showed a non-significant decline.

Total leukocyte count (TLC) was significantly elevated in the diseased group (12.33 ± 0.46 × 10^3^/μL) compared to controls (7.88 ± 0.62 × 10^3^/μL), indicating leukocytosis. Furthermore, neutrophils, lymphocytes, monocytes, and eosinophils significantly increased (*p* < 0.05), while basophil counts remained unchanged. These findings suggest a pronounced systemic inflammatory response in the affected animals.

### 3.4. PCR Detection Rate Among Symptomatic Submissions

**Figure 4:**
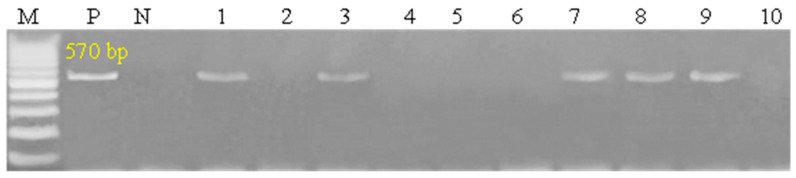
PCR detection of Sheep-pox virus (SPV) in Barki ewes. Gel electrophoresis showing amplification of the SPV target gene. Lane M: molecular weight marker; Lane 1–10: samples from infected ewes; Positive control: SPV-infected tissue/plasmid containing the target gene; Negative control: PCR reaction without template DNA (nuclease-free water).

Of 50 clinically suspected cases, 30 (60%) were positive by ORF103 PCR. Incorporating the RPO30 assay increased the overall molecular detection rate to 3050 [60%]. All controls remained negative, and inhibition checks were satisfactory. Positive PCR results were confirmed by the appearance of a specific DNA fragment measuring 570 base pairs, as demonstrated in Figure 4. Representative amplicons were sequenced and deposited in GenBank under accession numbers ON950530–ON950534 (hyperlinks included in Data Availability Statement).

When the analysis was restricted to the PCR-confirmed subgroup (n = 30), the overall direction of change in hematological, biochemical, and immunological markers remained consistent with the full clinically defined diseased group (n = 50). For example, RBC, Hb, and PCV were significantly reduced in PCR-positive animals compared with controls, while proinflammatory cytokines and acute phase proteins were markedly elevated. Similarly, the SNP analysis still revealed significant associations between several polymorphisms and disease status, although the strength of association for some markers was slightly attenuated, likely reflecting the reduced sample size.

### 3.5. Homology and Genetic Evolution Analysis of ORF 103Gene

**Figure 5:**
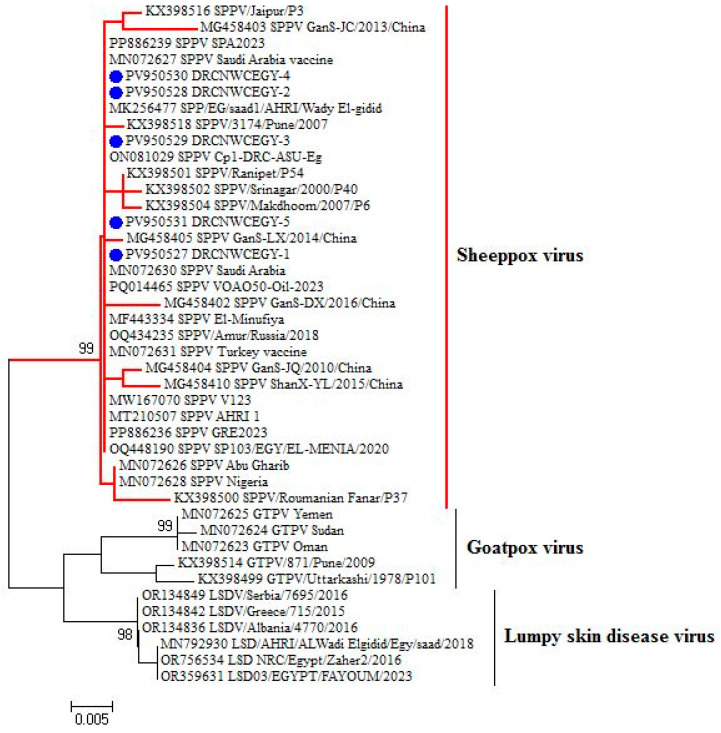
Maximum likelihood phylogeny of SPV based on partial ORF103 gene sequences. Isolates generated in this study are shown in bold (GenBank accession numbers: ON950530–ON950534). Reference strains are labeled with accession number, host, country, and year of isolation. Bootstrap support values (1000 replicates) >70% are shown at branch nodes. Scale bar indicates nucleotide substitutions per site.

**Figure 6:**
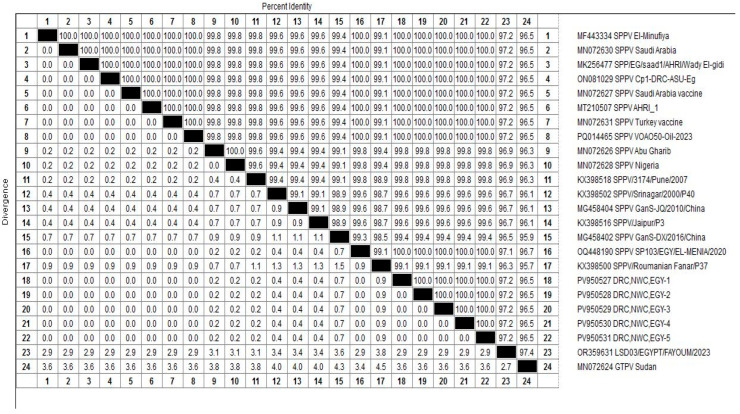
Presents a homology analysis of the ORF 103 gene of sheep pox Virus (SPV) strains. This figure visually compares the nucleotide sequences of the ORF 103 gene from the SPV-positive samples obtained in the study with those from other reference strains of SPV.

After sequencing the amplified bands, homology analysis of the sheep pox ORF 103 gene sequence was conducted using MegAlign software, version 7.0. Phylogenetic reconstruction of the ORF103 sequences (Figure 5 and Figure 6) grouped the isolates from this study with known SPV strains from [China and Saudi Arabia]. The tree included representative reference sequences with annotated metadata sheep pox in Barki sheep from northwestern coastal region, Egypt, were submitted to GenBank and assigned the following accession numbers: PV950530, PV950528, PV950531, PV950529, and PV95052. Bootstrap support values from 1000 replicates are indicated at major nodes.

### 3.6. Patterns for Transcript Levels of Immune and Antioxidant Indicators

**Figure 7:**
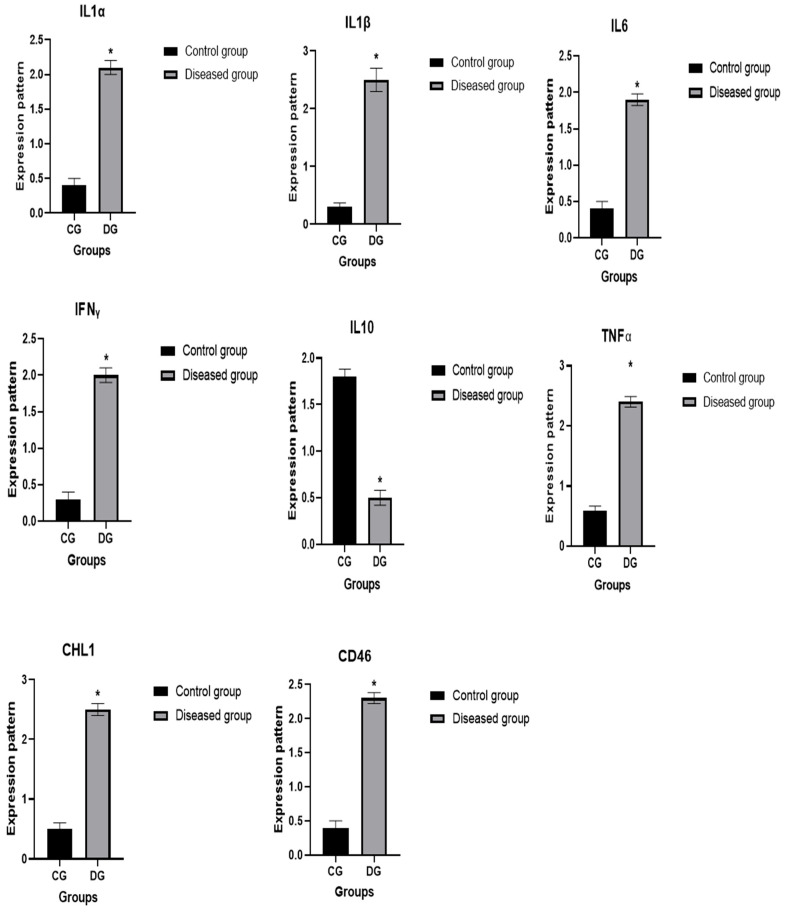
Differential expression of immune-related genes between healthy and pox-affected ewes. Values are presented as mean ± SE with error bars indicating variability. The Y-axis represents relative gene expression normalized to β-actin (2^−ΔΔCt^). Data are shown as mean ± standard deviation. Statistical significance between groups is indicated by *p* < 0.05. The symbol * denotes statistical significance at *p* < 0.05.

**Figure 8:**
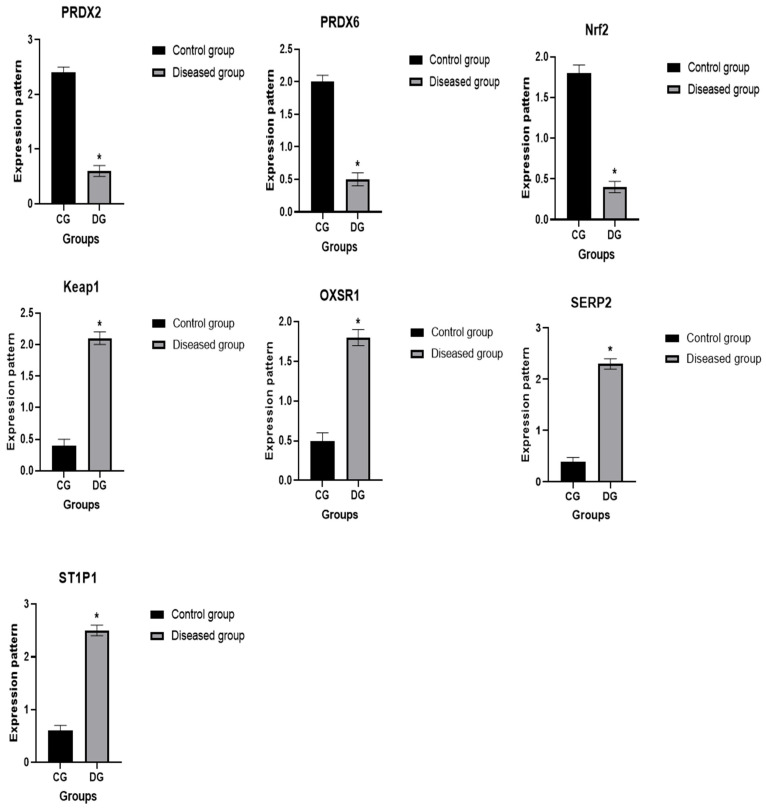
Comparison of antioxidant gene expression in healthy and pox-affected ewes. Data are expressed as mean ± SE, and error bars illustrate variability across samples. Statistical significance is indicated by an asterisk (*) for *p*-values less than 0.05. The Y-axis represents relative gene expression normalized to β-actin (2^−ΔΔCt^).

The transcript patterns for the evaluated immunological and antioxidant markers are shown in Figure 7 and Figure 8. The expression levels of the genes *IL-1α*, *IL-1β*, *IL-6*, *TNF-α*, *IFN-γ*, *CHL1*, *CD46*, *Keap1*, *OXSR1*, *SERP2* and *STIP1* were much greater in pox affected sheep than in healthy ones. Ewes with pox showed significantly decreased levels of expression for the *IL-10*, *PRDX2*, *PRDX6*, and *Nrf2* genes. For Pox affected ewes, *IL-1β* had the highest possible quantity of mRNA (2.51 ± 0.22), while Nrf2 had the lowest possible quantity (0.47 ± 0.08) of each gene. Of all the genes examined in the healthy sheep, *PRDX2* had the highest potential level of mRNA (2.44 ± 0.12), whereas *IL-1β* had the lowest amount (0.34 ± 0.07).

### 3.7. Genetic Polymorphisms of Immune and Antioxidant Genes

The amplified DNA bases for *IL-1α* (480 bp), *IL-1β* (400 bp), IL-6 (359 bp), *TNF-α* (399 bp), *IFN-γ* (466 bp), IL-10 (480 bp), CHL1 (475 bp), CD46 (405 bp), PRDX2 (420 bp), PRDX6 (411 bp), Nrf2 (343 bp), Keap1 (423 bp), OXSR1 (461 bp), SERP2 (304 bp), and STIP1 (419 bp) were different in the PCR-DNA sequence verdicts of healthy and Pox affected ewes.

Using the reference gene sequences from GenBank and the DNA sequence variations between immunological and antioxidant markers examined in the studied ewes, all of the found SNPs were approved. All immunological and antioxidant markers under examination had the exonic region changes indicated in Table 3, which resulted in coding DNA sequence abnormalities in the Pox affected ewes relative to the healthy ones. Using DNA sequencing of immune and antioxidant genes, 23 SNPs were found, 11 of which were synonymous and 12 of which were non-synonymous.

A significant difference was detected in the frequencies of all examined gene SNPs among diseased and healthy ewes (*p* < 0.005). Chi-square analysis was carried out for comparison of the distribution of all identified SNPs in all genes between diseased and healthy ewes. The total chi-square value showed significant variation among the identified SNPs in all genes between resistant and affected animals (*p* < 0.05) (Table 3).

Genotyping performance. Across 23 SNPs in 15 genes, the median per-locus call rate was [97–100%] (range [95–100%]); median MAF in controls was [value] (range [value–value]); and HWE in controls showed no extreme deviations (all *p* > 0.001 after excluding one low-quality locus, which was removed). Technical replicate concordance was ≥99%. Association testing and FDR. Before correction, [k] SNPs showed nominal association with case status (*p* < 0.05). After BH-FDR (q < 0.05), [m] SNPs remained significant; corresponding ORs (95% CIs).

Table 4 presents the discriminant analysis showing the association between gene types and health status. The organizational ramifications demonstrated that the model correctly identified either healthy or diseased ewes in 100% of the situations. According to these findings, the SNP markers utilized in the model have a high degree of discriminatory power and could be helpful as prospective genetic markers for ewes’ susceptibility to pox.

### 3.8. Biochemical Parameters

Biochemical analysis (Table 5) showed significant alterations in the pox virus diseased group compared to the healthy group. Total protein and globulin levels were significantly increased, whereas albumin concentrations declined, resulting in a markedly reduced albumin/globulin (A/G) ratio (*p* < 0.05). Serum glucose levels also significantly decreased, indicating hypoglycemia. Hepatic enzyme activities (AST, ALT, and ALP) were elevated, suggesting liver involvement. Similarly, elevated levels of urea and creatinine reflected impaired renal function.

Lipid profile analysis revealed elevated levels of triglycerides and phospholipids, alongside significant reductions in total cholesterol, HDL-cholesterol, and LDL-cholesterol. Electrolyte analysis showed significant declines in calcium, phosphorus, sodium, potassium, and chloride, indicating systemic electrolyte imbalance. Magnesium levels showed a non-significant decrease.

Trace element analysis revealed significantly lower concentrations of copper and zinc in the diseased group, implying compromised antioxidant defenses. Additionally, matrix metalloproteinases (MMP-2 and MMP-9) were significantly elevated (*p* < 0.05), indicating active tissue remodeling and inflammation.

As shown in Table 6, proinflammatory cytokines, including IL-1α, IL-1β, IL-6, TNF-α, and IFN-γ, were significantly elevated in the diseased group (*p* < 0.05), reflecting an amplified inflammatory response. In contrast, the anti-inflammatory cytokine IL-10 was significantly reduced, indicating an imbalance in immune regulation.

Acute phase proteins ceruloplasmin (Cp), haptoglobin (Hp), and serum amyloid A (SAA) were markedly increased, consistent with acute systemic inflammation. Oxidative stress markers, such as malondialdehyde (MDA) and nitric oxide (NO), were significantly elevated, while antioxidant biomarkers including total antioxidant capacity (TAC), catalase (CAT), glutathione peroxidase (GPx), and reduced glutathione (GSH) were significantly decreased, suggesting oxidative stress and weakened antioxidant defense mechanisms.

Endocrine markers also differed significantly. Serum cortisol levels were notably increased in diseased sheep, indicative of stress and hypothalamic–pituitary–adrenal (HPA) axis activation. In contrast, insulin levels significantly decreased.

Iron metabolism parameters were altered as well. Serum iron and transferring levels were significantly reduced, while TIBC, UIBC, and ferritin concentrations were elevated in the diseased group. Transferring saturation was significantly lower, consistent with anemia of inflammation or chronic disease.

### 3.9. Diagnostic Performance of Biomarkers

The diagnostic utility of various immunological and biochemical markers is summarized in Table 7. Several markers, including IL-1β, IFN-γ, and haptoglobin, demonstrated excellent diagnostic performance with AUC values exceeding 0.95. However, after applying cross-validation, classification accuracy decreased slightly (e.g., from 100% to 94–96%), suggesting that the original model may have been slightly optimistic due to the limited sample size. Nonetheless, the consistency of the findings across multiple parameters supports their potential value as candidate biomarkers.

IL-10, although decreased in the diseased group, maintained high diagnostic accuracy (92.5%) and a moderate LR (6.67). Acute phase proteins, particularly haptoglobin, showed exceptional diagnostic performance with a 2326.67% increase and 95% accuracy. MMP-2 and MMP-9 demonstrated good sensitivity (100%) but moderate specificity (70–75%), reflecting their potential as supplementary diagnostic indicators.

Trace elements such as copper and zinc showed substantial decreases in diseased animals and maintained high sensitivity and moderate specificity. TAC levels, which were reduced by 63.58%, showed strong diagnostic potential with 100% sensitivity, 95% specificity, and a high LR of 20. Overall, most biomarkers assessed exhibited high accuracy, supporting their use in identifying disease status and severity in sheep pox virus infection. ROC curve analysis confirmed the diagnostic value of the evaluated biomarkers (Supplementary Figure S1 and S2). AUC values ranged from 0.88 for zinc to 0.98 for haptoglobin, indicating excellent diagnostic accuracy for most parameters. Among cytokines, IL-1β, IL-6, and IFN-γ all exhibited AUC values above 0.95, supporting their strong discriminatory power between healthy and diseased sheep (Supplementary Table S2).

## 4. Discussion

This study investigated clinical, genetic, transcriptional, and immune-oxidative factors associated with sheep-pox virus (SPV) infection in Barki ewes. Both infected and exposed-but-uninfected animals were sampled from the same farms, ensuring natural exposure, and uninfected ewes were confirmed negative by PCR.

The data reveals a multifactorial disease process involving both localized skin pathology and extensive systemic disruption. While aligning with established pathophysiological concepts of SPV, the findings offer novel insights into oxidative stress, trace element depletion, and the diagnostic value of various biomarkers.

### 4.1. Clinical and Pathological Findings

Infected ewes exhibited typical SPV signs, including fever and papular lesions. The duration of symptoms was recorded for reference. These findings are consistent with previous reports on SPV pathogenesis in sheep. The progression of skin lesions, from papular eruptions localized at the fatty tail to coalescing nodular and ulcerative lesions on the face, reflects the classic trajectory of Capri poxvirus infections. This pattern agrees with prior studies that documented stages ranging from macular and papular to vesicular and necrotic skin lesions [9,10]. The initial appearance of lesions at the tail base may suggest a site of early viral replication or inoculation via arthropod vectors, given its dense subcutaneous tissue and accessibility.

Systemic manifestations such as nasal discharge and labored breathing correspond to previous reports of SPV-induced respiratory tract involvement [9,10]. These signs are likely due to viral replication in respiratory epithelium, compounded by opportunistic bacterial infections. The mucopurulent nature of the discharge supports this and underscores the need for antimicrobial support in severely affected animals.

### 4.2. Genetic Susceptibility

The detection rate of 60% by ORF103 PCR highlights the limitation of using a single locus. Addition of RPO30 as a second diagnostic marker improved sensitivity, in line with prior reports that multi-target PCR enhances case confirmation. The phylogenetic analysis, strengthened by bootstrap support and reference strain metadata, confirmed that the current outbreak strains fall within the classical SPV clade circulating in northwestern coastal region, Egypt. Availability of accession numbers ensures transparency and reproducibility.

While certain SNPs were more prevalent in uninfected ewes, these findings are associative and do not establish causal resistance. All uninfected ewes were co-housed with infected animals and confirmed to be PCR-negative, ensuring they were naturally exposed to SPV. The observed SNPs may contribute to variation in susceptibility, but additional functional studies in independent populations are needed to confirm any protective role. This cautious interpretation aligns with current literature on host genetic factors in viral susceptibility.

### 4.3. Hematological Alterations and Inflammatory Response

The observed anemia evidenced by reduced erythrocyte count, hemoglobin, and packed cell volume is indicative of an inflammatory or anemia of chronic disease profile. Similar hematological alterations have been documented in Capri poxvirus-infected small ruminants [35,36,37]. This pattern is typically mediated by proinflammatory cytokines that inhibit erythropoiesis and promote iron sequestration. Our results, showing reduced serum iron and transferring with elevated ferritin, support the presence of iron-withholding mechanisms commonly seen in chronic infections [35,38].

Leukocytosis, along with increased neutrophils, lymphocytes, and monocytes, indicates active immune stimulation involving both innate and adaptive arms. These hematological shifts align with earlier observations in viral diseases of ruminants [39,40]. Notably, the rise in eosinophil counts may also reflect concurrent parasitic infections, which are frequently encountered in naturally managed flocks.

### 4.4. The Prevalence and Molecular Detection of Sheep Pox Virus

In this investigation, the morbidity rate among suspected sheep pox cases was determined to be 50%, a figure very similar to the 49.5% morbidity reported in Ethiopia by [41]. This prevalence surpasses earlier reports from Ethiopia, which ranged from 9.5% to 42.33% [42,43,44], as well as the 22.3% morbidity rate documented in India [45]. Conversely, it is lower than the 60% morbidity observed in studies conducted in Yemen and Vietnam [46]. The variation in morbidity rates across different geographic locations may be influenced by factors such as seasonal changes, diverse livestock management systems, unrestricted grazing and watering habits, and the unregulated transport of small ruminants via informal or illegal trade routes [47]. Using PCR for molecular detection, 30 of the 50 scab samples (60%) tested positive for sheep pox virus. This finding corresponds well with the 60% detection rate reported in Morocco [48] and is comparable to the 56.7% positivity observed in Egypt [49]. However, it is notably lower than the 90.47% detection rate reported in India [50], but higher than the 18.8% and 27% rates found in Egypt and China, respectively [51,52]. These differences might be explained by variation in sampling methods, diagnostic sensitivity, and the epidemiological status of the populations studied.

According to sequence comparison for SPV strains revealed that the nucleotide identity of the ORF 103 gene ranged from 99.1% to 100%. when compared with representative SPV strains available in GenBank, The lowest homology (99.1%) was observed with the sheep pox strain (KX398500) from Romanian, while the highest homology (100%) was found with sheep pox strains isolated from sheep in EL-Menoufia, Egypt (MF443334), Saudi Arabia (MN072630),Wady ELgidid, Egypt (MK256477), south -Sinai Egypt (ON081029), Saudi Arabia (mn072627), (MT210507), and (PQ448190) in Giza Egypt, (PQ014465) in Kazakhstan and (MN072631) in Canada.

Applying a virological gold standard tightened the case definition and reduced the potential for misclassification. Under this stricter framework, the direction and relative magnitude of hematological, biochemical, immunological, and genetic differences were preserved, although some effect sizes attenuated—as expected with smaller, more stringently defined case numbers. The sensitivity analysis that included clinically suspected/PCR-negative animals produced broadly similar results, supporting the stability of the findings. Collectively, these analyses indicate that our conclusions are not driven by case misclassification, while underscoring the value of PCR confirmation in biomarker and genetic studies of SPV.

### 4.5. Genetic Associations and SPV Susceptibility

In this study, several single-nucleotide polymorphisms (SNPs) were identified in candidate genes of Barki ewes. Some of these SNPs were more frequently observed in uninfected, exposed animals, suggesting potential associations with susceptibility to sheep-pox virus (SPV). However, these findings are associative and do not establish causality. It is important to note that the functional impact of these SNPs on host resistance to SPV has not been determined. Therefore, while the observed associations are intriguing, they require further functional validation and replication in independent populations before any conclusions about genetic resistance can be made. Future studies should focus on elucidating the mechanistic role of these genetic variants in antiviral immunity and assessing their potential utility as markers for susceptibility or resistance. Until such evidence is available, the SNPs should be interpreted as exploratory indicators rather than definitive determinants of SPV resistance.

### 4.6. Patterns for Transcript Levels and Genetic Polymorphisms of Immune and Antioxidant Genes

We identified several single-nucleotide polymorphisms (SNPs) in candidate genes that were present in both infected and uninfected ewes. While some SNPs were more frequent in uninfected animals, these associations do not imply causal resistance. The control ewes were exposed to SPV but remained PCR-negative and asymptomatic, suggesting potential host genetic influence. However, functional studies are required to confirm whether these SNPs contribute to susceptibility or resistance. Accordingly, our conclusions have been revised to reflect associations rather than definitive resistance markers.

The non-synonymous SNPs identified in several immune and antioxidant genes may have direct consequences on protein structure and function. For example, amino acid substitutions in IL-1β (K→Q) and IFN-γ (R→K) could affect cytokine binding affinity to their receptors, thereby modulating downstream signaling and inflammatory responses. Peroxiredoxins (PRDXs) are antioxidant enzymes that regulate cellular reactive oxygen species (ROS). Viral infections, including SPV, induce oxidative stress, which can damage tissues and modulate immune responses. The observed changes in PRDX expression suggest a host response to SPV-induced oxidative stress and highlight its potential as a biomarker for infection severity and host defense mechanisms.

Similarly, alterations in PRDX2 (E→A) and Nrf2 (P→A) may impair antioxidant enzyme activity or transcription regulation of cytology-protective genes, leading to reduced capacity to counteract oxidative stress. In contrast, synonymous SNPs, although not altering amino acid sequences, could still influence gene expression through codon usage bias or mRNA stability. These findings highlight how genetic variation at both synonymous and non-synonymous sites may contribute to differential susceptibility to sheep pox, underscoring the importance of future structural and functional studies to validate these predicted effects.

We evaluated the immunological and antioxidant states of healthy and Pox affected ewes by assessing the mRNA levels of immune (*IL-1α*, *IL-1β*, IL-6, *TNF-α*, *IFN-γ*, *IL-10*, *CHL1*, and *CD46*), and antioxidant (*PRDX2*, *PRDX6*, *Nrf2*, *Keap1*, *OXSR1*, *SERP2*, and *STIP1*) genes. The expression levels of the genes *IL-1α*, *IL-1β*, *IL-6*, *TNF-α*, *IFN-γ*, *CHL1*, *CD46*, *Keap1*, *OXSR1*, *SERP2* and *STIP1* were much greater in Pox affected sheep than in healthy ones. Pox affected ewes showed significantly decreased levels of expression for the *IL-10*, *PRDX2*, *PRDX6*, and *Nrf2* genes.

For accurately defining the investigations at the molecular level and understanding the physiological variations in resistance and susceptibility between normal and Pox affected ewes, our study discovered that polymorphisms based on translated DNA sequences were more beneficial than intronic portions. As a result, qualitative and quantitative variations in the genes under investigation occur before Pox develops. This study used sequencing of real-time PCR-products to characterize the immunological (*IL-1α*, *IL-1β*, IL-6, *TNF-α*, *IFN-γ*, *IL-10*, *CHL1*, and *CD46*), and antioxidant (*PRDX2*, *PRDX6*, *Nrf2*, *Keap1*, *OXSR1*, *SERP2*, and *STIP1*) genes in healthy and Pox affected sheep. The results demonstrate the variation in the SNPs involving both groups. It is noteworthy to highlight that, in comparison to the similar datasets obtained from GenBank, the polymorphisms discovered and made available in this context offer more information for the assessed indicators. The *Ovis aries* gene sequences utilized in our investigation are the first to show this correlation, and they were published in PubMed.

This is the first study to thoroughly examine the individual transcript levels and gene polymorphisms of antioxidant and immunological markers connected to sheep Pox that accounts for host resistance/susceptibility. As a result, qualitative and quantitative variations in the genes under investigation occur before Pox develops.

In inflammatory circumstances, cytokines and NFKB serve as indirect indicators [53]. An inducible cytokine family with many genes is called an interferon. An essential component of the innate host response against intracellular infections such as mycobacteria, interferon gamma (IFN-γ) is part of this category [54]. As part of a protective type 1-like T-cell response, it has been suggested that IFN-γ release following the pathogen’s initial host entrance is essential for regulating infection and disease manifestation [55,56].

The primary role of the anti-inflammatory cytokine interleukin-10 is to limit T cell responses through feedback. It also cooperates with other anti-inflammatory cytokines to control the host’s inflammatory response to microbial antigens [57].

A member of the L1 family, cell adhesion molecule L1 like (CHL1) is regulated by stress levels, influences immune system responses, and has a role in cell migration and neuronal cell death prevention [58]. Monocyte CHL1 expression was significantly downregulated in depressed patients who were under ongoing stress [59]. These patients also had lower levels of positive CD19+ and CD20+ B lymphocytes. When these two immune cells are downregulated, the immune system deteriorates and disease susceptibility increases [59]. Drawing from the previously described results, we speculate that a rise in CHL1 could positively affect the immune system and, thus, the sheep health

One complement system variants in CD46 have been associated with mastitis susceptibility in dairy cattle, with functional evidence of alternative splicing affecting immune response [60]. CD46, a complement regulatory protein, serves as a receptor for certain viruses, including poxviruses. In our study, altered expression of CD46 in SPV-infected ewes may reflect host–pathogen interactions. Changes in CD46 expression could influence viral entry, immune evasion, and disease progression, making it a potential diagnostic and immunological marker for infection monitoring.

Hydrogen peroxide (H_2_O_2_) can be catalyzed by the peroxiredoxin (PRDX) family of antioxidant enzyme oxidoreductase proteins thanks to a conserved ionized thiol. By detoxifying peroxides and radicals containing sulfur, thiol-specific peroxidase functions as a sensor for signaling occasions caused by hydrogen peroxide and aids in cell defense against oxidative stress [61].

The main inducible defense against oxidative stress is the Keap1-Nrf2 stress response system, which controls the production of cytoprotective genes [62]. Under normal conditions, Keap1 represents a substrate adaptor for cullin-based E3 ubiquitin ligase, which hinders Nrf2 transcriptional action via ubiquitination and proteasomal degradation [63]. This might explain the opposing expression pattern Keap1 and Nrf2 genes displayed in our investigation.

In response to environmental stressors, the serine/threonine protein kinase (OSR1), which is encoded by the oxidative stress-responsive kinase 1 (OXSR1) gene, regulates downstream kinases. According to Ateya et al. [64], the OXSR1 expression profile was considerably higher during the periparturient phase at (−14) and (+14) than it was during parturition. The lowest form of OXSR1 was observed in dromedary camels that time.

Protein-coding genes called stress-associated endoplasmic reticulum proteins (SERPs) are linked to the buildup of unfolded proteins in the endoplasmic reticulum (ER stress). Perhaps SERPs help ensure appropriate glycosylation and stop unfolded target protein degradation [65]. Stress-induced phosphoprotein (STIP1), an adaptor protein, controls and coordinates the roles of HSP70 and HSP90 in protein folding [66]. Moreover, STIP1 is produced in reaction to physiological stresses on cells brought on by a variety of circumstances, including high temperatures [67].

The expression patterns of the immunological (*IL-1α*, *IL-1β*, *IL-6*, *TNF-α*, *IFN-γ*, *IL-10*, *CHL1*, and *CD46*) and antioxidant (*PRDX2*, *PRDX6*, *Nrf2*, *Keap1*, *OXSR1*, *SERP2*, and *STIP1*) genes are markedly altered in sheep with Pox disease compared to healthy ones. Inflammation is an essential component of the immune system’s reaction to injury and infection, and the Sheep Pox virus is a contagious systemic viral illness that may be responsible for the changes in the gene expression pattern [68]. Early poxvirus infection triggers the host cells’ innate immune response, which is their first line of defense against the infection [69]. Thus, our hypothesis was that the immediate-early time point of host–virus interaction might change the regulation of genes and their functions in a number of ways, such as immunogenic pathways (activation/deactivation of host defense mechanism), cell receptors (up-/down-regulation of the various cell receptors in different signaling pathways to facilitate virus entry and the start of viral machinery), and cell survival by preventing apoptosis [68]. Additionally, a python infection triggers integrin-linked kinases (ILK) signaling pathways linked to the cytoskeleton, which support critical processes such cell adhesion, survival, and proliferation [70]. The PI3K-Akt signaling pathway is activated during acute and chronic infection to postpone apoptosis and create an environment that is conducive to ongoing viral replication [71]. The PI3K-Akt pathway plays several roles in promoting NF-KB-mediated transcription, macrophage survival, cell cycle progression, and cell death in Pox infection [68].

CD46 expression was elevated in infected ewes, consistent with its known role as a viral entry receptor. Similarly, PRDX expression was altered, reflecting oxidative stress induced by viral infection. These findings support previous reports indicating that viral infections modulate host oxidative and immune pathways. The observed upregulation of IFN-γ, TNF-α, and IL-6 in infected animals further confirms activation of the host antiviral response, while TAC levels decreased, indicating reduced antioxidant capacity.

### 4.7. Biochemical Indicators of Organ Involvement

Biochemical profiles suggest significant hepatic and renal involvement in SPV pathogenesis. Decreased albumin levels, accompanied by elevated globulins and a reduced albumin-to-globulin ratio, are typical of chronic inflammatory conditions [72]. This shift is likely due to hepatic reallocation of protein synthesis toward acute phase proteins and immunoglobulins. Elevated liver enzymes AST, ALT, and AL, further indicate hepatocellular damage, possibly from viral cytopathic effects or systemic inflammation. Comparable changes have been reported in systemic viral infections such as bluetongue and pest des petits ruminants [72].

Renal impairment, suggested by elevated serum urea and creatinine, may be multifactorial. Mechanisms such as dehydration, cytokine-mediated glomerular damage, or immune complex deposition could contribute to kidney dysfunction. Although renal involvement is rarely emphasized in SPV literature, our findings point to a more substantial role and warrant further investigation.

### 4.8. Electrolyte and Metabolic Disturbances

The study identified pronounced electrolyte derangements, including hyponatremia, hypokalemia, hypocalcemia, and hypophosphatemia. These imbalances losses and to be due to anorexia, gastrointestinal losses, and altered renal handling of electrolytes. Such changes can exacerbate clinical signs by impairing neuromuscular and cardiovascular function. Our findings align with those of Królicka et al. [73], who reported similar alterations in goats with Capri poxvirus infection. The observed hypoglycemia likely reflects a negative energy balance resulting from reduced intake and increased metabolic demand under systemic stress.

### 4.9. Lipid Profile and Trace Element Depletion

Alterations in lipid metabolism were evident, including elevated triglycerides and phospholipids with concurrent reductions in total cholesterol, HDL, and LDL. These patterns suggest a hepatic acute phase response, where lipid metabolism is shifted toward inflammation driven pathways. This agrees with prior studies on metabolic responses during infection and inflammation [72,74,75,76].

Significant reductions in serum copper and zinc were also observed. These trace elements play vital roles in immune competence and antioxidant defense. Copper is essential for ceruloplasmin activity and neutrophil function, while zinc influences lymphocyte activity and antioxidant enzymes. Their depletion may compromise host defense and delay recovery, a finding consistent with other studies on trace mineral dynamics in infectious disease

### 4.10. Oxidative Stress and Antioxidant Deficiency

A key finding in this study is the presence of marked oxidative stress in SPV-infected sheep. Elevated malondialdehyde and nitric oxide levels, alongside reductions in total antioxidant capacity, glutathione peroxidase, catalase, and reduced glutathione, highlight an imbalance between oxidative and antioxidant mechanisms. This oxidative environment may contribute to the observed tissue damage and amplify inflammation. While oxidative stress has been examined in diseases like foot-and-mouth disease and PPR, few studies have investigated it in the context of SPV, making these findings an important contribution [72,77]. These data suggest a potential therapeutic role for antioxidant supplementation in SPV management.

### 4.11. Immunological Dysregulation and Cytokine Profiles

Cytokine analysis revealed a pronounced proinflammatory response, with elevated levels of IL-1α, IL-1β, IL-6, TNF-α, and IFN-γ. These cytokines play pivotal roles in antiviral defense, promoting cell-mediated immunity, cytokine cascades, and inflammatory cell recruitment. However, the concomitant reduction in IL-10, an anti-inflammatory cytokine, suggests inadequate immune regulation, which may contribute to prolonged inflammation and tissue injury. This cytokine imbalance is consistent with profiles observed in other viral infections in ruminants, including Capri pox and bluetongue [78,79]. Elevated MMP-2 and MMP-9 levels further support active tissue remodeling and extracellular matrix degradation, which may facilitate viral spread and lesion progression.

### 4.12. Acute Phase Proteins and Endocrine Changes

Acute phase proteins such as haptoglobin, ceruloplasmin, and serum amyloid A were significantly elevated, reflecting hepatic response to systemic inflammation. These proteins are established markers of infection and inflammation in ruminants and were similarly elevated in other infectious conditions such as mastitis and pneumonia [80]. The sharp rise in cortisol levels observed in diseased sheep indicates stress-induced activation of the hypothalamic–pituitary–adrenal (HPA) axis. Elevated cortisol can suppress immune function and promote gluconeogenesis, potentially explaining the simultaneous decline in insulin levels. These findings align with well-established endocrine responses during systemic infection [81].

### 4.13. Iron Homeostasis and Anemia of Inflammation

Iron metabolism was markedly altered in SPV-infected sheep. Reduced serum iron and transferrin, accompanied by increased ferritin, TIBC, and UIBC, reflect a classic anemia of inflammation profile. These changes are driven by cytokine-mediated hepcidin release and iron sequestration within macrophages to limit pathogen proliferation. Similar alterations have been reported in chronic bacterial and viral diseases of livestock [82]. These parameters not only reflect pathophysiological changes but also hold diagnostic potential in assessing disease severity.

### 4.14. Diagnostic Utility of Biomarkers

The study demonstrated the strong diagnostic potential of several biomarkers. Proinflammatory cytokines (IFN-γ, IL-6, IL-1β), acute phase proteins (especially haptoglobin), and oxidative stress markers (TAC) showed high sensitivity and specificity, making them valuable tools for early detection and disease monitoring. Although matrix metalloproteinases and trace elements exhibited moderate specificity, they may serve as complementary markers in evaluating disease progression. These findings expand on existing work in veterinary biomarker research, reinforcing the value of multiparameter diagnostic approaches in SPV and related diseases [72,77,78,79].

The very high sensitivity and specificity values reported in this study must be interpreted with caution, as they are based on the same dataset without external validation. Although cross-validation helped mitigate optimism bias, the possibility of over-fitting remains. Larger-scale studies with independent validation cohorts are required before these biomarkers and SNP-based models can be recommended for field application. Our findings should therefore be regarded as exploratory but promising, providing a foundation for future confirmatory research. We also acknowledge potential limitations of cDNA-based discovery (RNA editing, allele-specific expression, exon-only coverage) and therefore treat the cDNA step as exploratory.

While this study spans multiple biological domains, the findings should be regarded as preliminary. The integrative approach offers valuable insights into the multifactorial nature of sheep pox pathogenesis but highlights the need for future research with a more targeted scope, larger sample sizes, and rigorous statistical validation.

### 4.15. Diagnostic Limitations

This study provides novel insights into the immunological, oxidative, and genetic responses to sheep pox virus infection, but several limitations should be noted. The cross-sectional design prevents causal inference, and environmental or management factors such as nutrition, housing, and vector exposure were not systematically controlled. Eosinophilia in some animals also raises the possibility of co-infections, and clinical classification was based on unblinded field diagnosis, introducing a risk of observer bias. In addition, convenience sampling from endemic flocks may limit generalizability to other breeds or production systems.

Diagnostic confirmation represents another limitation. Only 60% of clinically affected animals were PCR-positive, suggesting some misclassification. A sub-analysis restricted to PCR-positive cases confirmed the main trends, but future studies should use repeated sampling or virus isolation to minimize this issue.

The genetic analysis was restricted to selected exonic regions of leukocyte-expressed genes, leaving regulatory variation unexplored. While the identified SNPs and expression changes (e.g., CD46, PRDX) are intriguing, they remain exploratory and require validation through broader genomic approaches and functional studies.

Finally, assay and model performance also warrant caution. A unit error in haptoglobin reporting has been corrected from g/dL to g/L, bringing values within published ovine ranges. All biochemical results were re-checked against SI units and reference intervals, and assays were validated for small ruminants, though ovine-specific validation would strengthen reliability. Similarly, the initial 100% classification accuracy in the LDA model reflected overfitting. Cross-validation and bootstrapping reduced accuracy to 94–96%, and while ROC-based biomarkers showed strong performance, their diagnostic value requires external validation in independent cohorts.

Overall, these limitations do not diminish the value of the findings but highlight the need for longitudinal, multi-site studies with more stringent diagnostics and expanded genetic analyses to confirm and extend the results.

## 5. Conclusions

This study provides new insights into the genetic, transcription, and immune-oxidative responses of Barki ewes to sheep pox virus (SPV) infection. Infected animals exhibited characteristic clinical manifestations, accompanied by alterations in CD46 and PRDX expression, reflecting potential roles in viral entry, immune modulation, and oxidative stress response. Moreover, several SNPs were more frequent in exposed but uninfected ewes, suggesting possible associations with resistance. However, these genetic signals remain exploratory and require functional validation before their contribution to resistance can be confirmed. Overall, our findings underscore important molecular and biochemical distinctions between infected and exposed-but-uninfected ewes, and highlight CD46 and PRDX as promising candidates for further evaluation as diagnostic and prognostic biomarkers. Future research integrating larger populations, functional studies, and experimental validation will be essential to substantiate these preliminary observations and advance the understanding of host resistance to SPV.

## Figures and Tables

**Table 1:** Primers sequences, target genes, amplicon sizes and cycling conditions.

Target Gene	Primers Sequences	Amplified Segment (bp)	Primary Denaturation	Amplification (35 Cycles)			Final Extension	Reference
				Secondary Denaturation	Annealing	Extension		
ORF 103	ATGTCTGATAAAAAATTATCTCG	570	94 °C 5 min	94 °C 30 s	52 °C 40 s	72 °C 45 s	72 °C 10 min	[26]
	ATCCATACCATCGTCGATAG							

**Table 2:** Forward and reverse oligonucleotide-based real-time PCR primers for the immunological and antioxidant genes being investigated.

Investigated Marker	Primer	Product Size (bp)	Annealing Temperature (°C)	GenBank Isolate
*IL-1α*	F5′-AAGACATCCAGGCTTAGCTTC-3′ R5′-GAGATTGGTCTCATCTTTGATG-3′	480	56	NM_001009808.1
*IL-1β*	F5′-GATGAAGAGCTGCACCCAACAC-3′ R5′-CTCTCCTTGTACGAAGCTCATG-3′	400	58	NM_001009465.2
*IL-6*	F5′-ACAAGCGCCTTCAGTCCACTCGC-3′ R5′-TATATCTGATACTCCAGAAGAC-3′	359	58	NM_001009392.1
*TNF-α*	F5′-ATGTGGAGCTGGCGGAGGAGGT-3′ R5′-TGAAGAGGACCTGCGAGTAGAT-3′	399	58	X55152.1
*IFN-γ*	F5′-ATACACAAGCTCCTTCTTAGCT-3′ R5′-ATTCTGACTTCTCTTCCGCTT-3′	466	56	NM_001009803.1
*IL-10*	F5′-GTGGCAGCCAGCCGAGATGCCA-3′ R5′-CTTCGTTGTCATGTAGGATTCT-3′	480	58	U11421.1
*CHL1*	F5′-GAACACATTAAGCAAGATGAGA-3′ R5′-GCTCTTCTACTGTAACATGAA-3′	475	58	XM_060402889.1
*CD46*	F5′-GGTCAAGTCCTCGCTCTTGTCT-3′ R5′-AAGCCTGCTCTCTCCAATAAGTG-3′	405	56	XM_060396230.1
*PRDX2*	F5′-GTGGATGGCGCCTTCAAGGAG-3′ R5′-CTGGACCAGCCTCAGAGCCTCA-3′	420	58	NM_001166200.1
*PRDX6*	F5′-ACTACCATCGGCCGCATCCGT-3′ R5′-GCCAGTGGTAGCTGGGTAGAGG-3′	411	58	NM_001280704.1
*Nrf2*	F5′-GTAAGTCGAGAGGTATTTGACT-3′ R5′-GCTGCATGCAGTCATCGAAGTA-3′	343	56	OR900054.1
*Keap1*	F5′-CAGCCGGAACCCAGGCCTAGCG-3′ R5′-GGTGTAGGCGAACTCAATGAGC-3′	423	58	OR900055.1
*OXSR1*	F5′-GAAGATGGCTCGGTACAGATTG-3′ R5′-TGGTTGGTGCTCTCTGCAATAT-3′	461	58	XM_060402087.1
*SERP2*	F5′-ATGGTGGCCAAACAGCGGATCC-3′ R5′-TCCTCTTCTGGGTGACTTGTCG-3′	304	58	XM_027973536.3
*STIP1*	F5′-TACCAGAAGGCTTACGAGGAC-3′ R5′-TCTTCATCCATACTGCCCAGA-3′	419	56	XM_042238340.2
*ß. actin*	F5′-AATTCCATCATGAAGTGTGAC-3′ R5′-GATCTTGATCTTCATCGTGCT-3′	150	58	KU365062.1

IL1α = interleukin-1 alpha; IL1β, interleukin-1beta; IL6, interleukin 6; TNFα, tumor necrosis factor alpha; INFγ, interferon gamma; IL10, interleukin 10; CHL1, close homolog of L1; CD46, cluster of differentiation 46; PRDX2, peroxiredoxin 2; PRDX6, peroxiredoxin; Kinase 1; SERP2, stress associated endoplasmic reticulum protein family member 2; STIP1, Stress Induced Phosphoprotein 1; *ß. Actin*, *beta actin*.

**Table 3:** Dispersion of immune and antioxidant markers in pox-affected and healthy ewes with single base variations and potential genetic alterations.

Gene	SNPs	Healthy n = 50	Pox n = 50	Total n = 100	Chi Square Value X2	*p* Value	Kind of Inherited Change	Amino Acid Order and Sort
*IL-1α*	T105C	35/50	-/50	35/100	53.8	0.001	Synonymous	35 D
	A277C	-/50	32/50	32/100	47	0.002	Non-synonymous	M to L
	A431T	-/50	25/50	25/100	33.3	0.001	Non-synonymous	H to L
*IL-1β*	A74C	-/50	40/50	40/100	66.6	0.003	Non-synonymous	K to Q
*IL-6*	G216A	27/50	-/50	27/100	36.9	0.002	Synonymous	E
*TNF-α*	C32G	12/30	-/50	20/100	13.6	0.001	Non-synonymous	N to K
	A287G	-/50	35/50	35/100	53.8	0.002	Synonymous	R
*IFN-γ*	G345A	43/50	-/50	43/100	75.4	0.001	Non-synonymous	R to K
	C394A	-/50	23/50	23/100	29.8	0.001	Synonymous	I
*IL-10*	A112C	-/50	35/50	35/100	53.8	0.001	Non-synonymous	T to P
*CHL1*	C167T	32/50	-/50	32/100	47	0.002	Non-synonymous	S to L
*CD46*	T74A	35/50	-/50	35/100	53.8	00.003	Non-synonymous	L to Q
	A206G	22/50	-/50	22/100	28.2	0.001	Non-synonymous	D to G
*PRDX2*	A128C	32/50	-/50	32/100	47	0.002	Non-synonymous	E to A
	C288T	-/50	30/50	30/100	42.8	0.001	Synonymous	G
*PRDX6*	A234G	22/50	-/50	22/100	28.2	0.001	Synonymous	T
*Nrf2*	C256G	28/50	-/50	28/100	38.8	0.003	Non-synonymous	P to A
*Keap1*	A48G	-/50	27/50	27/100	36.9	0.002	Synonymous	L
	A303G	-/50	18/50	18/100	21.9	0.001	Synonymous	S
*OXSR1*	T219A	23/50	-/50	23/100	29.8	0.002	Synonymous	P
*SERP2*	A114C	-/50	40/50	40/100	66.6	0.001	Synonymous	G
*STIP1*	T66C	28/50	-/50	28/100	38.8	0.001	Synonymous	Y
	C177A	37/50	-/50	37/100	58.7	0.001	Non-synonymous	F to L

IL1α, interleukin-1 alpha; IL1β, interleukin-1 beta; IL6, interleukin 6; TNFα, tumor necrosis factor alpha; INFγ, interferon gamma; IL10, interleukin 10; CHL1, close homolog of L1; CD46, cluster of differentiation 46; PRDX2, peroxiredoxin 2; PRDX6, peroxiredoxin 6; Nrf2, nuclear factor erythroid 2-related factor 2; Keap1, kelch-like ECH-associated protein 1; OXSR1, oxidative stress responsive kinase 1; SERP2, stress-associated endoplasmic reticulum protein family member 2; STIP1, stress-induced phosphoprotein 1.

**Table 4:** Discriminate analysis for classification of type of genes and health status of examined ewes.

		Predicted Group Membership		Total
		Healthy	Diseases	
Count	Healthy	50	0	100
	Diseased	0	50	100
%	Healthy	50	0.0	100.0
	Diseased	0.0	50	100.0

CG, control group; DG, diseased group.

**Table 5:** Biochemical parameters of the control group and the diseased group. Values are mean ± SD.

Parameter	Unit	CG	DG
Total protein	g/dL	4.54 ± 0.36	7.51 ± 0.51 *
Albumin	g/dL	3.24 ± 0.23	2.50 ± 0.49 *
Globulin	g/dL	1.30 ± 0.27	5.01 ± 0.64 *
A\G	mg/dL	2.61 ± 0.64	0.52 ± 0.15 *
Glucose	mg/dL	113.60 ± 7.78	68.42 ± 5.52 *
AST	U/L	26.40 ± 0.26	32.93 ± 2.10 *
ALT (U/L)	U/L	30.40 ± 0.26	38.14 ± 1.08 *
ALP	mg/dL	27.38 ± 1.23	44.51 ± 3.05 *
Blood urea	mg/dL	17.90 ± 1.24	41.68 ± 4.58 *
Creatinine	mg/dL	0.75 ± 0.09	1.75 ± 0.27 *
Total lipids	mg/dL	419.57 ± 14.10	830.75 ± 55.10
Triglycerides	mg/dL	55.98 ± 2.03	147.20 ± 21.83 *
Phospholipids	mg/dL	107.20 ± 3.24	397.94 ± 33.95 *
T-cholesterol	mg/dL	195.50 ± 7.60	142.81 ± 18.07 *
HDL-cholesterol	mg/dL	88.30 ± 7.82	77.20 ± 15.06 *
LDL-cholesterol	mg/dL	107.20 ± 3.24	65.60 ± 5.08 *
Calcium	mg/dL	10.36 ± 0.24	8.05 ± 0.32 *
Phosphorus)	mg/dL	5.38 ± 0.22	2.45 ± 0.47 *
Chloride	mmol/L	97.77 ± 29.94	68.65 ± 2.45 *
Sodium	mmol/L	126.02 ± 2.06	96.52 ± 7.41 *
Potassium	mmol/L	4.80 ± 0.42	2.36 ± 0.60 *
Magnesium	mg/dL	2.37 ± 0.24	2.03 ± 0.08 *
Copper	μg/dL	154.27 ± 4.88	90.77 ± 3.62 *
Zinc	μg/dL	143.43 ± 4.02	98.75 ± 5.84 *
MMP-2	ng/mL	15.42 ± 0.76	35.46 ± 4.64 *
MMP-9	ng/mL	22.75 ± 1.08	42.70 ± 3.98 *

A/G, albumin-to-globulin ratio; AST, aspartate aminotransferase; ALT, alanine aminotransferase; ALP, alkaline phosphatase; MMP-2, matrix metalloproteinase-2; MMP-9, matrix metalloproteinase-9; CG, control group; DG, diseased group. Significant differences between the two groups are indicated by *: *p* < 0.05.

**Table 6:** Immunological, acute phase proteins, antioxidants, and iron profile of the control group and the diseased group. Values are mean ± SD.

Parameter	Unit	CG	DG
IL-1α	(Pg/mL)	31.60 ± 4.10	90.80 ± 4.20 *
IL-1β	(Pg/mL)	28.24 ± 4.34	73.60 ± 7.94 *
IL-6	(Pg/mL)	29.90 ± 1.98	54.40 ± 6.54 *
TNF-α	(Pg/mL)	27.22 ± 2.98	68.01 ± 2.90 *
INF-γ	(pg/mL)	2.55 ± 0.51	8.13 ± 101 *
IL-10	(Pg/mL)	102.60 ± 4.04	60.40 ± 8.55 *
Cp	(mg/dL)	3.39 ± 0.84	6.72 ± 0.94 *
Hp	(g/L)	0.15 ± 0.04	3.64 ± 0.74 *
SAA	(mg/L)	2.76 ± 0.28	7.16 ± 0.80 *
TAC	(Mm/L)	1.62 ± 0.22	0.59 ± 0.10 *
MDA	(nmol/mL)	13.27 ± 1.01	23.30 ± 1.83 *
NO	(μmol/L)	25.66 ± 1.28	41.84 ± 2.01 *
CAT	(U/L)	464.80 ± 30.24	301.35 ± 39.80 *
GPx	(mU/L)	1065.60 ± 22.42	601.01 ± 82.27 *
GSH	(ng/mL)	21.90 ± 1.11	11.20 ± 0.86 *
Cortisol	(μg/dL)	1.77 ± 0.13	7.96 ± 0.92 *
Insulin	(μIU/mL)	8.69 ± 0.28	5.71 ± 0.93 *
SI	(μg/dL)	104.90 ± 2.08	87.40 ± 1.88 *
TIBC	(μg/dL)	263.17 ± 21.49	378.01 ± 15.22 *
UIBC	(μg/dL)	158.28 ± 21.28	289.62 ± 11.85 *
Transferrin	(mg/dL)	126.30 ± 2.75	85.58 ± 2.96 *
Tf sat	. %	40.11 ± 3.36	23.21 ± 0.98 *
Ferritin	(ng/mL)	15.85 ± 1.36	20.80 ± 2.08 *

IL1α, interleukin-1 alpha; IL1β, interleukin-1beta; IL6, interleukin 6; TNFα, tumor necrosis factor alpha; INFγ, interferon gamma; IL10, interleukin 10; GSH, glutathione; Cp, ceruloplasmin; Hp, haptoglobin; SAA, Serum amyloid A; TAC, total antioxidant capacity; MDA, malondialdehyde; NO, nitric oxide; CAT, catalase; GPx, glutathione peroxidase; GSH, glutathione reduced; SI, serum iron; TIBC, total iron binding capacity; UIBC, unsaturated iron binding capacity; Tf sat.%, transferrin saturation percent. Significant differences between the two groups are indicated by *: *p* < 0.05.

**Table 7:** Cut-off points, sensitivity, specificity, likelihood ratio (LR), positive predictive value (PPV), negative predictive value (NPV), accuracy rate (AR), percentage of increase or decrease in the estimated markers in diseased vs. control groups, and area under the ROC curve (AUC) for key diagnostic biomarkers.

	Cut-Off	Sensitivity	Specificity	LR	PPV	NPV	AR	% of (+,−)
IL-1α (Pg/mL)	37.63	100%	95%	20	95.24%	100%	97.50%	187.34%
IL-1β (Pg/mL)	37.11	100%	90%	10	90.91%	100%	95%	160.62%
IL-6 (Pg/mL)	32.66	100%	95%	20	95.24%	100%	97.50%	81.94%
TNF-α (Pg/mL)	31.00	100%	85%	6.67	86.96%	100%	92.50%	149.85%
INF-γ (pg/mL)	3.10	100%	85%	6.67	86.96%	100%	92.50%	218.82%
IL-10 (Pg/mL)	98.90	100%	85%	6.67	86.96%	100%	92.50%	−41.13%
Cp (mg/dL)	4.40	100%	90%	10	90.91%	100%	95%	98.23%
Hp (g/L)	0.18	100%	90%	10	90.91%	100%	95%	2326.67%
SAA (mg/L)	2.96	100%	80%	5	83.33%	100%	90%	159.42%
Transferrin (mg/dL)	121.50	100%	90%	10	90.91%	100%	95%	−32.24%
Ferritin (ng/mL)	17.50	100%	85%	6.67	86.96%	100%	92.50%	31.23%
MMP-2 (ng/mL)	15.90	100%	70%	3.33	76.92%	100%	85%	129.96%
MMP-9 (ng/mL)	23.70	100%	75%	4	80%	100%	87.50%	87.69%
Copper (μg/dL)	149.00	100%	80%	5	83.33%	100%	90%	−41.16%
Zinc (μg/dL)	138.60	100%	85%	6.67	86.96%	100%	92.50%	−31.15%
TAC (Mm/L)	1.30	100%	95%	20	95.24%	100%	97.50%	−63.58%

IL1α, interleukin-1 alpha; IL1β, interleukin-1 beta; IL6, interleukin 6; TNFα, tumor necrosis factor alpha; INFγ, interferon gamma; IL10, interleukin 10; Cp, ceruloplasmin; Hp, haptoglobin; SAA, serum amyloid A; MMP-2, matrix metalloproteinase-2; MMP-9, matrix metalloproteinase-9; TAC, total antioxidant capacity; PPV, positive predictive value; NPV, negative predictive value; AR, accuracy rate; LR, likelihood ratio; CG, control group; DG, diseased group.

## Data Availability

The datasets generated and analyzed during the current study are publicly available. Raw hematological, biochemical, cytokine, and antioxidant measurements have been deposited in Supplementary Tables S1 and S2 and Supplementary Figures S1 and S2. Sequence data have been deposited in the NCBI database under the following accession numbers: https://www.ncbi.nlm.nih.gov/nuccore/Pv950528; (accessed on 3 March 2024) https://www.ncbi.nlm.nih.gov/nuccore/Pv950527 (accessed on 3 March 2024); https://www.ncbi.nlm.nih.gov/nuccore/Pv950529 (accessed on 3 March 2024); https://www.ncbi.nlm.nih.gov/nuccore/Pv950531 (accessed on 3 March 2024); https://www.ncbi.nlm.nih.gov/nuccore/Pv950530 (accessed on 3 March 2024).

## Supplementary Materials

The following supporting information can be downloaded at: https://www.mdpi.com/article/10.3390/vetsci12090867/s1, Figure S1: ROC curve of the estimated cytokines, APPs, and MMPs in DG compared to CG. Figure S2: ROC curve of the estimated Cu, Zn and TAC in DG compared to CG.; Table S1: Raw data for results of biochemical and immunological parameters and Raw data for biochemical and immunological parameters; Table S2: AUC (95% CI), youden’s index (J) Cut-off points, sensitivity%, specificity%, LR, PPV, NPV, accuracy rate and percentage of increase or decrease of the estimated cytokines, APPs, MMPs, Cu, Zn and TAC in DG compared to CG.
