# Supplementary material for: Sheep Pox Susceptibility: Role of Genetic Variants, Gene Expression, and Immune-Oxidative Markers
Source: Vet Sci. 2025 Sep 8;12(9):867. doi: 10.3390/vetsci12090867 (PMC12474403; doi:10.3390/vetsci12090867)
Supplement: Supplementary file 1 [file vetsci-12-00867-s001.zip › vetsci-3839312- Supplementrt Table S2 (Cut off table) (1).pdf]

**Table 8:** **AUC** (95% CI), **youden`s index (J)** Cut-off points, sensitivity%, specificity%, LR, PPV, NPV, accuracy rate and percentage of increase or decrease of the estimated cytokines, APPs, MMPs, Cu, Zn and TAC in DG compared to CG.

| | **AUC** | **Youden`s index (J)** | **Cut-off** | **Sensitivity** | **Specificity** | **LR** | **PPV** | **NPV** | **AR** | **% of (+,-)** |
|---|---|---|---|---|---|---|---|---|---|---|
| IL-1α (Pg/ml) | 1 | 0.95 | 37.63 | 100% | 95% | 20 | 95.24% | 100% | 97.50% | 187.34% |
| IL-1β (Pg/ml) | 1 | 0.90 | 37.11 | 100% | 90% | 10 | 90.91% | 100% | 95% | 160.62% |
| IL-6 (Pg/ml) | 1 | 0.95 | 32.66 | 100% | 95% | 20 | 95.24% | 100% | 97.50% | 81.94% |
| TNF-α (Pg/ml) | 1 | 0.85 | 31.00 | 100% | 85% | 6.67 | 86.96% | 100% | 92.50% | 149.85% |
| INF-γ(Pg/ ml) | 1 | 0.85 | 3.10 | 100% | 85% | 6.67 | 86.96% | 100% | 92.50% | 218.82% |
| IL-10 (Pg/ml) | 1 | 0.85 | 98.90 | 100% | 85% | 6.67 | 86.96% | 100% | 92.50% | -41.13% |
| Cp (mg/ml) | 1 | 0.90 | 4.40 | 100% | 90% | 10 | 90.91% | 100% | 95% | 98.23% |
| Hp (g/L) | 1 | 0.90 | 0.18 | 100% | 90% | 10 | 90.91% | 100% | 95% | 2326.67% |
| SAA (µg/ml) | 1 | 0.80 | 2.96 | 100% | 80% | 5 | 83.33% | 100% | 90% | 159.42% |
| Transferrin (mg/dl) | 1 | 0.90 | 121.50 | 100% | 90% | 10 | 90.91% | 100% | 95% | -32.24% |
| Ferritin (ng/mL) | 0.98 | 0.85 | 17.50 | 100% | 85% | 6.67 | 86.96% | 100% | 92.50% | 31.23% |
| MMP-2 (ng/ml) | 1 | 0.70 | 15.90 | 100% | 70% | 3.33 | 76.92% | 100% | 85% | 129.96% |
| MMP-9 (ng/ml) | 1 | 0.75 | 23.70 | 100% | 75% | 4 | 80% | 100% | 87.50% | 87.69% |
| Copper (µg/dl) | 1 | 0.80 | 149.00 | 100% | 80% | 5 | 83.33% | 100% | 90% | -41.16% |
| Zinc (µg/dl) | 1 | 0.85 | 138.60 | 100% | 85% | 6.67 | 86.96% | 100% | 92.50% | -31.15% |
| TAC (mmol/L) | 1 | 0.95 | 1.30 | 100% | 95% | 20 | 95.24% | 100% | 97.50% | -63.58% |

**$0 < J < 0.3$** → Poor test, **$0.3 \leq J < 0.6$** → Moderate test, **$0.6 \leq J < 0.8$** → Good test, **$0.8 \leq J \leq 1$** → Excellent test. AUC = 0.5–0.65 (useless marker), AUC = 0.7–0.85 (good marker), AUC = 0.86–1 (with satisfactory sensitivity and specificity: excellent marker). LR= 0.5-5: low; LR=5-10: moderate; LR>10: high.
