# Supplementary material for: Sheep Pox Susceptibility: Role of Genetic Variants, Gene Expression, and Immune-Oxidative Markers
Source: Vet Sci. 2025 Sep 8;12(9):867. doi: 10.3390/vetsci12090867 (PMC12474403; doi:10.3390/vetsci12090867)
Supplement: Supplementary file 1 [file vetsci-12-00867-s001.zip › vetsci-3839312- Supplementry Table S3 (Raw Data).pdf]

GROUP	RBCS	HB	HCT	MCh	MCv	MCHC	c	bwt
1	10	8	27	27	8	29.63	40	40
1	10.88	8	28	25.74	7.35	28.57	39.8	38
1	9.88	9	30	30.36	9.11	30	40	4
1	10.66	8	28	26.27	7.5	28.57	39.8	42
1	10.54	9	30	28.46	8.54	30	40.2	38.5
1	10	8.5	28	28	8.5	30.36	39.6	39
1	10	8	27	27	8	29.63	40	40
1	10.88	8	28	25.74	7.35	28.57	39.8	38
1	9.88	9	30	30.36	9.11	30	40	4
1	10.66	8	28	26.27	7.5	28.57	39.8	42
1	10.54	9	30	28.46	8.54	30	40.2	38.5
1	10	8.5	28	28	8.5	30.36	39.6	39
1	10	8	27	27	8	29.63	40	40
1	10.88	8	28	25.74	7.35	28.57	39.8	38
1	9.88	9	30	30.36	9.11	30	40	4
1	10.66	8	28	26.27	7.5	28.57	39.8	42
1	10.54	9	30	28.46	8.54	30	40.2	38.5
1	10	8.5	28	28	8.5	30.36	39.6	39
1	10	8	27	27	8	29.63	40	40
1	10.88	8	28	25.74	7.35	28.57	39.8	38
1	10	8	27	27	8	29.63	40	40
1	10.88	8	28	25.74	7.35	28.57	39.8	38
1	9.88	9	30	30.36	9.11	30	40	4
1	10.66	8	28	26.27	7.5	28.57	39.8	42
1	10.54	9	30	28.46	8.54	30	40.2	38.5
1	10	8.5	28	28	8.5	30.36	39.6	39
1	10	8	27	27	8	29.63	40	40
1	10.88	8	28	25.74	7.35	28.57	39.8	38
1	9.88	9	30	30.36	9.11	30	40	4
1	10.66	8	28	26.27	7.5	28.57	39.8	42
1	10.54	9	30	28.46	8.54	30	40.2	38.5
1	10	8.5	28	28	8.5	30.36	39.6	39
1	10	8	27	27	8	29.63	40	40
1	10.88	8	28	25.74	7.35	28.57	39.8	38
1	9.88	9	30	30.36	9.11	30	40	4
1	10.66	8	28	26.27	7.5	28.57	39.8	42
1	10.54	9	30	28.46	8.54	30	40.2	38.5
1	10	8.5	28	28	8.5	30.36	39.6	39
1	10	8	27	27	8	29.63	40	40
1	10.88	8	28	25.74	7.35	28.57	39.8	38
1	10	8	27	27	8	29.63	40	40
1	10.88	8	28	25.74	7.35	28.57	39.8	38
1	9.88	9	30	30.36	9.11	30	40	4
1	10.66	8	28	26.27	7.5	28.57	39.8	42
1	10.54	9	30	28.46	8.54	30	40.2	38.5
1	10	8.5	28	28	8.5	30.36	39.6	39
1	10	8	27	27	8	29.63	40	40
1	10.88	8	28	25.74	7.35	28.57	39.8	38
1	9.88	9	30	30.36	9.11	30	40	4

1	10.66	8	28	26.27	7.5	28.57	39.8	42
2	12.88	13	39	30.28	10.09	33.33	37	50
2	12.98	13	39	30.05	10.02	33.33	37.8	52
2	12.96	12	39	30.09	9.26	30.77	38	48
2	12.94	12	39	30.14	9.27	30.77	37	50
2	12.92	12	39	30.19	9.29	30.77	37.8	49
2	12.5	11.5	38	30.4	9.2	30.26	38	50
2	12.2	11.4	37	30.33	9.34	30.81	37	52
2	12.2	11.2	37	30.33	9.18	30.27	37.8	48
2	12	11	36.5	30.42	9.17	30.14	38	50
2	12	11	36	30	9.17	30.56	37	49
2	12	11	36	30	9.17	30.56	37.8	50
2	12	11	36	30	9.17	30.56	38	52
2	11.98	10.88	36	30.05	9.08	30.22	37	48
2	11.94	10.73	36	30.15	8.99	29.81	37.8	50
2	11.94	10.7	36	30.15	8.96	29.72	38	49
2	11.89	10.58	36	30.28	8.9	29.39	37	50
2	11.83	10.55	37	31.28	8.92	28.51	37.8	52
2	11.59	10.5	37	31.92	9.06	28.38	38	48
2	11.88	10.5	37	31.14	8.84	28.38	37.5	50
2	12	10.44	37	30.83	8.7	28.22	37.6	49
2	12.88	13	39	30.28	10.09	33.33	37	50
2	12.98	13	39	30.05	10.02	33.33	37.8	52
2	12.96	12	39	30.09	9.26	30.77	38	48
2	12.94	12	39	30.14	9.27	30.77	37	50
2	12.92	12	39	30.19	9.29	30.77	37.8	49
2	12.5	11.5	38	30.4	9.2	30.26	38	50
2	12.2	11.4	37	30.33	9.34	30.81	37	52
2	12.2	11.2	37	30.33	9.18	30.27	37.8	48
2	12	11	36.5	30.42	9.17	30.14	38	50
2	12	11	36	30	9.17	30.56	37	49
2	12	11	36	30	9.17	30.56	37.8	50
2	12	11	36	30	9.17	30.56	38	52
2	11.98	10.88	36	30.05	9.08	30.22	37	48
2	11.94	10.73	36	30.15	8.99	29.81	37.8	50
2	11.94	10.7	36	30.15	8.96	29.72	38	49
2	11.89	10.58	36	30.28	8.9	29.39	37	50
2	11.83	10.55	37	31.28	8.92	28.51	37.8	52
2	11.59	10.5	37	31.92	9.06	28.38	38	48
2	11.88	10.5	37	31.14	8.84	28.38	37.5	50
2	12	10.44	37	30.83	8.7	28.22	37.6	49
2	12.88	13	39	30.28	10.09	33.33	37	50
2	12.98	13	39	30.05	10.02	33.33	37.8	52
2	12.96	12	39	30.09	9.26	30.77	38	48
2	12.94	12	39	30.14	9.27	30.77	37	50
2	12.92	12	39	30.19	9.29	30.77	37.8	49
2	12.5	11.5	38	30.4	9.2	30.26	38	50
2	12.2	11.4	37	30.33	9.34	30.81	37	52
2	12.2	11.2	37	30.33	9.18	30.27	37.8	48
2	12	11	36.5	30.42	9.17	30.14	38	50

2	12	11	36	30	9.17	30.56	37	49

Full Legend for the Table

GROUP – Experimental group identifier.

RBCs – Red Blood Cell count (×10^6/µL).

HB – Hemoglobin concentration (g/dL).

HCT – Hematocrit (%).

MCH – Mean Corpuscular Hemoglobin (pg).

MCV – Mean Corpuscular Volume (fL).

MCHC – Mean Corpuscular Hemoglobin Concentration (g/dL).

c – Control/condition value (context-dependent).

bwt – Body weight (g).

WBC – White Blood Cell count (×

n – Neutrophil count (% or absol

l – Lymphocyte count (% or abso

m – Monocyte count (% or absol

e – Eosinophil count (% or absolu

b – Basophil count (% or absolut

TP – Total Protein (g/dL).

Alb – Albumin (g/dL).

Glob – Globulin (g/dL).

A/G – Albumin/Globulin ratio.

Glucose – Blood glucose level (m

Urea – Blood urea (mg/dL).

Cr – Serum Creatinine (mg/dL).

ALT – Alanine Aminotransferase

GROUP
RBCS
HB
HCT
MCh
MCv
MCHC
c
bwt
WBC
n
l
m
e
b
TP
alb
glob
AG
GLUCOSE
UREA
CR
ALT
AST
ALP
TL
TRI
TC
LDLC
HDLC

	PHOSL
**AST** – Aspartate Aminotransfera	NNA
	k
**ALP** – Alkaline Phosphatase (U/L	CL
	P
**TL** – Total Lipids (mg/dL).	CA
	MGG
**TRI** – Triglycerides (mg/dL).	CU
	ZN
**TC** – Total Cholesterol (mg/dL).	TAC
	CAAT
**LDL-C** – Low-Density Lipoprotein	GPXX
	GSSH
**HDL-C** – High-Density Lipoprotei	NOO
	MDAA
**PHOSL** – Phospholipids (mg/dL).	COR
	INS
**Na** – Sodium (mmol/L).	CPP
	HPP
**K** – Potassium (mmol/L).	SAAA
	IL1
**Cl** – Chloride (mmol/L).	IL111
	IL6
**P** – Phosphorus (mg/dL).	TNF
	INF
**Ca** – Calcium (mg/dL).	IL10
	MMP2
**Mg** – Magnesium (mg/dL).	MMP9
	iron
**Cu** – Copper (µg/dL).	tibc
	uibc
**Zn** – Zinc (µg/dL).	tf
	feretei
**TAC** – Total Antioxidant Capacity	tfper

CAT – Catalase activity (U/mL).

GPx – Glutathione Peroxidase activity (U/mL).

GSH – Reduced Glutathione (µmol/L).

NO – Nitric Oxide (µmol/L).

MDA – Malondialdehyde (marker of lipid peroxidation, nmol/mL).

COR – Cortisol (µg/dL).

INS – Insulin (µU/mL).

CPP – C-Peptide (ng/mL).

HPP – Haptoglobin (mg/dL).

SAA – Serum Amyloid A (mg/L).

IL-1 – Interleukin-1 (pg/mL).

IL-11 – Interleukin-11 (pg/mL).

IL-6 – Interleukin-6 (pg/mL).

TNF – Tumor Necrosis Factor-α (pg/mL).

INF – Interferon-γ (pg/mL

WBC	n	l	m	e	b	TP	alb	glob
12	8.4	2.4	0.84	0.36	0	7.03	2.92	4.11
12.89	9.02	2.58	0.9	0.39	0.01	7.4	1.7	5.7
12.86	9	2.57	0.9	0.39	0	7.47	2.88	4.59
12.01	8.4	2.4	0.84	0.36	0.01	8	2.89	5.11
11.88	8.32	2.38	0.83	0.36	0	8.4	2.57	5.83
12	8.4	2.4	0.84	0.36	0	6.97	2.19	4.78
12.89	9.02	2.58	0.9	0.39	0.01	7.03	2.92	4.11
12.86	9	2.57	0.9	0.39	0	7.4	1.7	5.7
12.01	8.4	2.4	0.84	0.36	0.01	7.47	2.88	4.59
11.88	8.32	2.38	0.83	0.36	0	8	2.89	5.11
12	8.4	2.4	0.84	0.36	0	8.4	2.57	5.83
12.89	9.02	2.58	0.9	0.39	0.01	6.97	2.19	4.78
12.86	9	2.57	0.9	0.39	0	7.03	2.92	4.11
12.01	8.4	2.4	0.84	0.36	0.01	7.4	1.7	5.7
11.88	8.32	2.38	0.83	0.36	0	7.47	2.88	4.59
12	8.4	2.4	0.84	0.36	0	8	2.89	5.11
12.89	9.02	2.58	0.9	0.39	0.01	8.4	2.57	5.83
12.86	9	2.57	0.9	0.39	0	6.97	2.19	4.78
12.01	8.4	2.4	0.84	0.36	0.01	7.03	2.92	4.11
11.88	8.32	2.38	0.83	0.36	0	7.4	1.7	5.7
12	8.4	2.4	0.84	0.36	0	7.03	2.92	4.11
12.89	9.02	2.58	0.9	0.39	0.01	7.4	1.7	5.7
12.86	9	2.57	0.9	0.39	0	7.47	2.88	4.59
12.01	8.4	2.4	0.84	0.36	0.01	8	2.89	5.11
11.88	8.32	2.38	0.83	0.36	0	8.4	2.57	5.83
12	8.4	2.4	0.84	0.36	0	6.97	2.19	4.78
12.89	9.02	2.58	0.9	0.39	0.01	7.03	2.92	4.11
12.86	9	2.57	0.9	0.39	0	7.4	1.7	5.7
12.01	8.4	2.4	0.84	0.36	0.01	7.47	2.88	4.59
11.88	8.32	2.38	0.83	0.36	0	8	2.89	5.11
12	8.4	2.4	0.84	0.36	0	8.4	2.57	5.83
12.89	9.02	2.58	0.9	0.39	0.01	6.97	2.19	4.78
12.86	9	2.57	0.9	0.39	0	7.03	2.92	4.11
12.01	8.4	2.4	0.84	0.36	0.01	7.4	1.7	5.7
11.88	8.32	2.38	0.83	0.36	0	7.47	2.88	4.59
12	8.4	2.4	0.84	0.36	0	8	2.89	5.11
12.89	9.02	2.58	0.9	0.39	0.01	8.4	2.57	5.83
12.86	9	2.57	0.9	0.39	0	6.97	2.19	4.78
12.01	8.4	2.4	0.84	0.36	0.01	7.03	2.92	4.11
11.88	8.32	2.38	0.83	0.36	0	7.4	1.7	5.7
12	8.4	2.4	0.84	0.36	0	7.03	2.92	4.11
12.89	9.02	2.58	0.9	0.39	0.01	7.4	1.7	5.7
12.86	9	2.57	0.9	0.39	0	7.47	2.88	4.59
12.01	8.4	2.4	0.84	0.36	0.01	8	2.89	5.11
11.88	8.32	2.38	0.83	0.36	0	8.4	2.57	5.83
12	8.4	2.4	0.84	0.36	0	6.97	2.19	4.78
12.89	9.02	2.58	0.9	0.39	0.01	7.03	2.92	4.11
12.86	9	2.57	0.9	0.39	0	7.4	1.7	5.7
12.01	8.4	2.4	0.84	0.36	0.01	7.47	2.88	4.59

11.88	8.32	2.38	0.83	0.36	0	8	2.89	5.11
8.85	6.64	1.77	0.35	0.09	0	4.88	3.2	1.68
8.85	6.64	1.77	0.35	0.09	0	4.24	3.44	0.8
8.55	6.41	1.71	0.34	0.09	1	4.52	3.01	1.51
8.2	6.15	1.64	0.33	0.08	0	4.6	3.2	1.4
8.1	6.08	1.62	0.32	0.08	0	4.22	3.32	0.9
8	6	1.6	0.32	0.08	0	4.32	3.2	1.12
7.85	5.89	1.57	0.31	0.08	1	5.32	3.8	1.52
7.75	5.81	1.55	0.31	0.08	0	4.22	3.28	0.94
7.7	5.78	1.54	0.31	0.08	0	4.88	3.44	1.44
7.65	5.74	1.53	0.31	0.08	0	4.24	3	1.24
7.65	5.74	1.53	0.31	0.08	0	4.52	3.12	1.4
7.4	5.55	1.48	0.3	0.07	0	4.6	3.06	1.54
7.4	5.55	1.48	0.3	0.07	1	4.22	3.04	1.18
7.4	5.55	1.48	0.3	0.07	0	4.32	3.02	1.3
7.25	5.44	1.45	0.29	0.07	0	5.32	3.45	1.87
7.2	5.4	1.44	0.29	0.07	0	4.88	3.66	1.22
7.1	5.33	1.42	0.28	0.07	1	4.24	3.12	1.12
7	5.25	1.4	0.28	0.07	0	4.52	3.02	1.5
8.85	6.64	1.77	0.35	0.09	0	4.6	3.4	1.2
8.85	6.64	1.77	0.35	0.09	0	4.22	3.11	1.11
8.85	6.64	1.77	0.35	0.09	0	4.88	3.2	1.68
8.85	6.64	1.77	0.35	0.09	0	4.24	3.44	0.8
8.55	6.41	1.71	0.34	0.09	1	4.52	3.01	1.51
8.2	6.15	1.64	0.33	0.08	0	4.6	3.2	1.4
8.1	6.08	1.62	0.32	0.08	0	4.22	3.32	0.9
8	6	1.6	0.32	0.08	0	4.32	3.2	1.12
7.85	5.89	1.57	0.31	0.08	1	5.32	3.8	1.52
7.75	5.81	1.55	0.31	0.08	0	4.22	3.28	0.94
7.7	5.78	1.54	0.31	0.08	0	4.88	3.44	1.44
7.65	5.74	1.53	0.31	0.08	0	4.24	3	1.24
7.65	5.74	1.53	0.31	0.08	0	4.52	3.12	1.4
7.4	5.55	1.48	0.3	0.07	0	4.6	3.06	1.54
7.4	5.55	1.48	0.3	0.07	1	4.22	3.04	1.18
7.4	5.55	1.48	0.3	0.07	0	4.32	3.02	1.3
7.25	5.44	1.45	0.29	0.07	0	5.32	3.45	1.87
7.2	5.4	1.44	0.29	0.07	0	4.88	3.66	1.22
7.1	5.33	1.42	0.28	0.07	1	4.24	3.12	1.12
7	5.25	1.4	0.28	0.07	0	4.52	3.02	1.5
8.85	6.64	1.77	0.35	0.09	0	4.6	3.4	1.2
8.85	6.64	1.77	0.35	0.09	0	4.22	3.11	1.11
8.85	6.64	1.77	0.35	0.09	0	4.88	3.2	1.68
8.85	6.64	1.77	0.35	0.09	0	4.24	3.44	0.8
8.55	6.41	1.71	0.34	0.09	1	4.52	3.01	1.51
8.2	6.15	1.64	0.33	0.08	0	4.6	3.2	1.4
8.1	6.08	1.62	0.32	0.08	0	4.22	3.32	0.9
8	6	1.6	0.32	0.08	0	4.32	3.2	1.12
7.85	5.89	1.57	0.31	0.08	1	5.32	3.8	1.52
7.75	5.81	1.55	0.31	0.08	0	4.22	3.28	0.94
7.7	5.78	1.54	0.31	0.08	0	4.88	3.44	1.44

7.65	5.74	1.53	0.31	0.08	0	4.24	3	1.24

AG	GLUCOSE	UREA	CR	ALT	AST	ALP	TL	TRI
0.71	68	42.8	1.56	37.04	29.8	39.18	927	147
0.3	60	42.28	1.29	38.58	36.85	46.33	809.28	122
0.63	65.45	34.44	1.8	39.84	32.8	48.11	816.49	125
0.57	70	48.55	1.9	37.88	30.46	47.38	764	170
0.44	75	44.55	1.72	38.41	32.44	42.08	837	172
0.46	75	36.88	2.2	37.38	34.02	45.66	927	147
0.71	68	42.8	1.56	39.18	35.22	44.02	809.28	122
0.3	60	42.28	1.29	36.33	32.88	40.77	816.49	125
0.63	65.45	34.44	1.8	37.04	29.8	39.18	764	170
0.57	70	48.55	1.9	38.58	36.85	46.33	837	172
0.44	75	44.55	1.72	39.84	32.8	48.11	927	147
0.46	75	36.88	2.2	37.88	30.46	47.38	809.28	122
0.71	68	42.8	1.56	38.41	32.44	42.08	816.49	125
0.3	60	42.28	1.29	37.38	34.02	45.66	764	170
0.63	65.45	34.44	1.8	39.18	35.22	44.02	837	172
0.57	70	48.55	1.9	36.33	32.88	40.77	927	147
0.44	75	44.55	1.72	39.84	32.8	48.11	809.28	122
0.46	75	36.88	2.2	37.88	30.46	47.38	816.49	125
0.71	68	42.8	1.7	38.41	32.44	42.08	764	170
0.3	60	42.28	1.8	37.38	34.02	45.66	837	172
0.71	68	42.8	1.56	37.04	29.8	39.18	927	147
0.3	60	42.28	1.29	38.58	36.85	46.33	809.28	122
0.63	65.45	34.44	1.8	39.84	32.8	48.11	816.49	125
0.57	70	48.55	1.9	37.88	30.46	47.38	764	170
0.44	75	44.55	1.72	38.41	32.44	42.08	837	172
0.46	75	36.88	2.2	37.38	34.02	45.66	927	147
0.71	68	42.8	1.56	39.18	35.22	44.02	809.28	122
0.3	60	42.28	1.29	36.33	32.88	40.77	816.49	125
0.63	65.45	34.44	1.8	37.04	29.8	39.18	764	170
0.57	70	48.55	1.9	38.58	36.85	46.33	837	172
0.44	75	44.55	1.72	39.84	32.8	48.11	927	147
0.46	75	36.88	2.2	37.88	30.46	47.38	809.28	122
0.71	68	42.8	1.56	38.41	32.44	42.08	816.49	125
0.3	60	42.28	1.29	37.38	34.02	45.66	764	170
0.63	65.45	34.44	1.8	39.18	35.22	44.02	837	172
0.57	70	48.55	1.9	36.33	32.88	40.77	927	147
0.44	75	44.55	1.72	39.84	32.8	48.11	809.28	122
0.46	75	36.88	2.2	37.88	30.46	47.38	816.49	125
0.71	68	42.8	1.7	38.41	32.44	42.08	764	170
0.3	60	42.28	1.8	37.38	34.02	45.66	837	172
0.71	68	42.8	1.56	37.04	29.8	39.18	927	147
0.3	60	42.28	1.29	38.58	36.85	46.33	809.28	122
0.63	65.45	34.44	1.8	39.84	32.8	48.11	816.49	125
0.57	70	48.55	1.9	37.88	30.46	47.38	764	170
0.44	75	44.55	1.72	38.41	32.44	42.08	837	172
0.46	75	36.88	2.2	37.38	34.02	45.66	927	147
0.71	68	42.8	1.56	39.18	35.22	44.02	809.28	122
0.3	60	42.28	1.29	36.33	32.88	40.77	816.49	125
0.63	65.45	34.44	1.8	37.04	29.8	39.18	764	170

0.57	70	48.55	1.9	38.58	36.85	46.33	837	172
1.9	114	17.11	0.8	30.44	26.44	26.44	413.68	52.68
4.3	105	18.86	0.82	30.66	26.66	26.88	400	58.8
1.99	112	15.79	0.84	30.88	26.88	24.88	389.01	56.82
2.29	108	18.86	0.86	30.44	26.44	28.32	381.03	55.78
3.69	106	17.97	0.72	30.32	26.32	26.98	394.08	55.82
2.86	125	19.3	0.66	30.12	26.12	28.88	376.92	52.68
2.5	112	16.67	0.68	30.02	26.02	26.44	397.04	58.8
3.49	124	17.54	0.7	30.64	26.64	28.22	378.02	56.82
2.39	126	19.74	0.6	30.24	26.24	28.44	397.97	55.78
2.42	110	17.12	0.8	30.2	26.2	28.32	389.07	55.82
2.23	114	17.11	0.82	30.44	26.44	26.44	382.08	55.82
1.99	116	18.86	0.84	30.66	26.66	26.88	392.92	52.68
2.58	102	15.79	0.86	30.88	26.88	24.88	417.8	58.8
2.32	107	18.86	0.72	30.44	26.44	28.32	390.02	56.82
1.84	120	17.97	0.66	30.32	26.32	26.98	377.97	55.78
3	126	19.3	0.68	30.12	26.12	28.88	399.07	55.82
2.79	109	16.67	0.7	30.02	26.02	26.44	386.94	52.68
2.01	102	17.54	0.6	30.64	26.64	28.22	387.04	58.8
2.83	120	19.74	0.84	30.24	26.24	28.44	431.82	56.82
2.8	114	17.12	0.8	30.2	26.2	28.32	388.98	55.78
1.9	114	17.11	0.8	30.44	26.44	26.44	413.68	52.68
4.3	105	18.86	0.82	30.66	26.66	26.88	400	58.8
1.99	112	15.79	0.84	30.88	26.88	24.88	389.01	56.82
2.29	108	18.86	0.86	30.44	26.44	28.32	381.03	55.78
3.69	106	17.97	0.72	30.32	26.32	26.98	394.08	55.82
2.86	125	19.3	0.66	30.12	26.12	28.88	376.92	52.68
2.5	112	16.67	0.68	30.02	26.02	26.44	397.04	58.8
3.49	124	17.54	0.7	30.64	26.64	28.22	378.02	56.82
2.39	126	19.74	0.6	30.24	26.24	28.44	397.97	55.78
2.42	110	17.12	0.8	30.2	26.2	28.32	389.07	55.82
2.23	114	17.11	0.82	30.44	26.44	26.44	382.08	55.82
1.99	116	18.86	0.84	30.66	26.66	26.88	392.92	52.68
2.58	102	15.79	0.86	30.88	26.88	24.88	417.8	58.8
2.32	107	18.86	0.72	30.44	26.44	28.32	390.02	56.82
1.84	120	17.97	0.66	30.32	26.32	26.98	377.97	55.78
3	126	19.3	0.68	30.12	26.12	28.88	399.07	55.82
2.79	109	16.67	0.7	30.02	26.02	26.44	386.94	52.68
2.01	102	17.54	0.6	30.64	26.64	28.22	387.04	58.8
2.83	120	19.74	0.84	30.24	26.24	28.44	431.82	56.82
2.8	114	17.12	0.8	30.2	26.2	28.32	388.98	55.78
1.9	114	17.11	0.8	30.44	26.44	26.44	413.68	52.68
4.3	105	18.86	0.82	30.66	26.66	26.88	400	58.8
1.99	112	15.79	0.84	30.88	26.88	24.88	389.01	56.82
2.29	108	18.86	0.86	30.44	26.44	28.32	381.03	55.78
3.69	106	17.97	0.72	30.32	26.32	26.98	394.08	55.82
2.86	125	19.3	0.66	30.12	26.12	28.88	376.92	52.68
2.5	112	16.67	0.68	30.02	26.02	26.44	397.04	58.8
3.49	124	17.54	0.7	30.64	26.64	28.22	378.02	56.82
2.39	126	19.74	0.6	30.24	26.24	28.44	397.97	55.78

2.42	110	17.12	0.8	30.2	26.2	28.32	389.07	55.82

TC	LDLC	HDLC	PHOSL	NNA	k	CL	P	CA
165.3	70.22	95.08	449.4	83.71	2.01	71.86	2.5	8.37
153.06	70.44	82.62	381.16	94.88	1.98	66.72	1.85	8.47
133.67	68.05	65.62	424.15	101.01	2.5	65.28	2.01	7.7
114	58.88	55.12	366	98	1.88	70.42	3	7.88
148	60.44	87.56	369	105	3.45	68.98	2.88	7.82
165.3	70.22	95.08	449.4	83.71	2.01	71.86	2.5	8.37
153.06	70.44	82.62	381.16	94.88	1.98	66.72	1.85	8.47
133.67	68.05	65.62	424.15	101.01	2.5	65.28	2.01	7.7
114	58.88	55.12	366	98	1.88	70.42	3	7.88
148	60.44	87.56	369	105	3.45	68.98	2.88	7.82
165.3	70.22	95.08	449.4	83.71	2.01	71.86	2.5	8.37
153.06	70.44	82.62	381.16	94.88	1.98	66.72	1.85	8.47
133.67	68.05	65.62	424.15	101.01	2.5	65.28	2.01	7.7
114	58.88	55.12	366	98	1.88	70.42	3	7.88
148	60.44	87.56	369	105	3.45	68.98	2.88	7.82
165.3	70.22	95.08	449.4	83.71	2.01	71.86	2.5	8.37
153.06	70.44	82.62	381.16	94.88	1.98	66.72	1.85	8.47
133.67	68.05	65.62	424.15	101.01	2.5	65.28	2.01	7.7
114	58.88	55.12	366	98	1.88	70.42	3	7.88
148	60.44	87.56	369	105	3.45	68.98	2.88	7.82
165.3	70.22	95.08	449.4	83.71	2.01	71.86	2.5	8.37
153.06	70.44	82.62	381.16	94.88	1.98	66.72	1.85	8.47
133.67	68.05	65.62	424.15	101.01	2.5	65.28	2.01	7.7
114	58.88	55.12	366	98	1.88	70.42	3	7.88
148	60.44	87.56	369	105	3.45	68.98	2.88	7.82
165.3	70.22	95.08	449.4	83.71	2.01	71.86	2.5	8.37
153.06	70.44	82.62	381.16	94.88	1.98	66.72	1.85	8.47
133.67	68.05	65.62	424.15	101.01	2.5	65.28	2.01	7.7
114	58.88	55.12	366	98	1.88	70.42	3	7.88
148	60.44	87.56	369	105	3.45	68.98	2.88	7.82
165.3	70.22	95.08	449.4	83.71	2.01	71.86	2.5	8.37
153.06	70.44	82.62	381.16	94.88	1.98	66.72	1.85	8.47
133.67	68.05	65.62	424.15	101.01	2.5	65.28	2.01	7.7
114	58.88	55.12	366	98	1.88	70.42	3	7.88
148	60.44	87.56	369	105	3.45	68.98	2.88	7.82
165.3	70.22	95.08	449.4	83.71	2.01	71.86	2.5	8.37
153.06	70.44	82.62	381.16	94.88	1.98	66.72	1.85	8.47
133.67	68.05	65.62	424.15	101.01	2.5	65.28	2.01	7.7
114	58.88	55.12	366	98	1.88	70.42	3	7.88
148	60.44	87.56	369	105	3.45	68.98	2.88	7.82
165.3	70.22	95.08	449.4	83.71	2.01	71.86	2.5	8.37
153.06	70.44	82.62	381.16	94.88	1.98	66.72	1.85	8.47
133.67	68.05	65.62	424.15	101.01	2.5	65.28	2.01	7.7
114	58.88	55.12	366	98	1.88	70.42	3	7.88
148	60.44	87.56	369	105	3.45	68.98	2.88	7.82
165.3	70.22	95.08	449.4	83.71	2.01	71.86	2.5	8.37
153.06	70.44	82.62	381.16	94.88	1.98	66.72	1.85	8.47
133.67	68.05	65.62	424.15	101.01	2.5	65.28	2.01	7.7
114	58.88	55.12	366	98	1.88	70.42	3	7.88

148	60.44	87.56	369	105	3.45	68.98	2.88	7.82
161	200	54.76	102.24	127	4.98	106.88	5.5	10.42
181	160.2	72.18	104.82	125.46	4.58	104.32	5.32	10.68
171	161.19	58.34	108.66	128.15	4.55	106	5.44	10.32
163	162.25	54	105	126.58	5.22	108	5.02	10.22
175	163.26	62.2	108.8	125.44	4.26	110	5.62	10.2
159	165.24	44.78	110.22	124.3	4.62	110	5.5	10.12
173	165.24	66.76	102.24	121.26	5.27	10.44	5.32	10.6
161	160.2	52.18	104.82	128.26	4.98	107.22	5.44	10.44
181	161.19	68.34	108.66	127	4.58	108.02	5.02	10.32
171	162.25	62	105	125.46	4.55	102.6	5.62	10.02
163	163.26	50.2	108.8	128.15	5.22	104.32	5.44	10.88
175	165.24	60.78	110.22	126.58	4.26	106	5.02	10.68
159	200	52.76	102.24	125.44	4.62	108	5.62	10.32
173	160.2	64.18	104.82	124.3	5.27	110	5.5	10.22
161	161.19	48.34	108.66	121.26	4.98	112	5.44	10.2
181	162.25	72	105	128.26	4.58	10.44	5.02	10.12
171	163.26	58.2	108.8	127	4.55	107.22	5.62	10.6
163	165.24	48.78	110.22	125.46	5.22	108.02	5.5	10.44
175	200	58.76	112.24	128.15	4.26	108	5.32	10.32
173	160.2	56.56	112.44	126.58	5.62	108	5.22	10.02
161	200	54.76	102.24	127	4.98	106.88	5.5	10.42
181	160.2	72.18	104.82	125.46	4.58	104.32	5.32	10.68
171	161.19	58.34	108.66	128.15	4.55	106	5.44	10.32
163	162.25	54	105	126.58	5.22	108	5.02	10.22
175	163.26	62.2	108.8	125.44	4.26	110	5.62	10.2
159	165.24	44.78	110.22	124.3	4.62	110	5.5	10.12
173	165.24	66.76	102.24	121.26	5.27	10.44	5.32	10.6
161	160.2	52.18	104.82	128.26	4.98	107.22	5.44	10.44
181	161.19	68.34	108.66	127	4.58	108.02	5.02	10.32
171	162.25	62	105	125.46	4.55	102.6	5.62	10.02
163	163.26	50.2	108.8	128.15	5.22	104.32	5.44	10.88
175	165.24	60.78	110.22	126.58	4.26	106	5.02	10.68
159	200	52.76	102.24	125.44	4.62	108	5.62	10.32
173	160.2	64.18	104.82	124.3	5.27	110	5.5	10.22
161	161.19	48.34	108.66	121.26	4.98	112	5.44	10.2
181	162.25	72	105	128.26	4.58	10.44	5.02	10.12
171	163.26	58.2	108.8	127	4.55	107.22	5.62	10.6
163	165.24	48.78	110.22	125.46	5.22	108.02	5.5	10.44
175	200	58.76	112.24	128.15	4.26	108	5.32	10.32
173	160.2	56.56	112.44	126.58	5.62	108	5.22	10.02
161	200	54.76	102.24	127	4.98	106.88	5.5	10.42
181	160.2	72.18	104.82	125.46	4.58	104.32	5.32	10.68
171	161.19	58.34	108.66	128.15	4.55	106	5.44	10.32
163	162.25	54	105	126.58	5.22	108	5.02	10.22
175	163.26	62.2	108.8	125.44	4.26	110	5.62	10.2
159	165.24	44.78	110.22	124.3	4.62	110	5.5	10.12
173	165.24	66.76	102.24	121.26	5.27	10.44	5.32	10.6
161	160.2	52.18	104.82	128.26	4.98	107.22	5.44	10.44
181	161.19	68.34	108.66	127	4.58	108.02	5.02	10.32

171	162.25	62	105	125.46	4.55	102.6	5.62	10.02

MGG	CU	ZN	TAC	CAAT	GPXX	GSSH	NOO	MDAA
2.05	92.58	101.7	0.47	241	682	12.37	42.88	20.88
2.05	92.2	102.54	0.66	333.33	544	10.88	44.66	22.68
1.88	95.44	103.52	0.65	352.32	488	10.88	38.9	24.42
2.04	88.24	98	0.49	288	698	11.86	40.66	26.06
2.12	85.4	88	0.7	292.08	588	10	42.04	22.44
2.05	92.58	101.7	0.47	241	682	12.37	42.88	20.88
2.05	92.2	102.54	0.66	333.33	544	10.88	44.66	22.68
1.88	95.44	103.52	0.65	352.32	488	10.88	38.9	24.42
2.04	88.24	98	0.49	288	698	11.86	40.66	26.06
2.12	85.4	88	0.7	292.08	588	10	42.04	22.44
2.05	92.58	101.7	0.47	241	682	12.37	42.88	20.88
2.05	92.2	102.54	0.66	333.33	544	10.88	44.66	22.68
1.88	95.44	103.52	0.65	352.32	488	10.88	38.9	24.42
2.04	88.24	98	0.49	288	698	11.86	40.66	26.06
2.12	85.4	88	0.7	292.08	588	10	42.04	22.44
2.05	92.58	101.7	0.47	241	682	12.37	42.88	20.88
2.05	92.2	102.54	0.66	333.33	544	10.88	44.66	22.68
1.88	95.44	103.52	0.65	352.32	488	10.88	38.9	24.42
2.04	88.24	98	0.49	288	698	11.86	40.66	26.06
2.12	85.4	88	0.7	292.08	588	10	42.04	22.44
2.05	92.58	101.7	0.47	241	682	12.37	42.88	20.88
2.05	92.2	102.54	0.66	333.33	544	10.88	44.66	22.68
1.88	95.44	103.52	0.65	352.32	488	10.88	38.9	24.42
2.04	88.24	98	0.49	288	698	11.86	40.66	26.06
2.12	85.4	88	0.7	292.08	588	10	42.04	22.44
2.05	92.58	101.7	0.47	241	682	12.37	42.88	20.88
2.05	92.2	102.54	0.66	333.33	544	10.88	44.66	22.68
1.88	95.44	103.52	0.65	352.32	488	10.88	38.9	24.42
2.04	88.24	98	0.49	288	698	11.86	40.66	26.06
2.12	85.4	88	0.7	292.08	588	10	42.04	22.44
2.05	92.58	101.7	0.47	241	682	12.37	42.88	20.88
2.05	92.2	102.54	0.66	333.33	544	10.88	44.66	22.68
1.88	95.44	103.52	0.65	352.32	488	10.88	38.9	24.42
2.04	88.24	98	0.49	288	698	11.86	40.66	26.06
2.12	85.4	88	0.7	292.08	588	10	42.04	22.44
2.05	92.58	101.7	0.47	241	682	12.37	42.88	20.88
2.05	92.2	102.54	0.66	333.33	544	10.88	44.66	22.68
1.88	95.44	103.52	0.65	352.32	488	10.88	38.9	24.42
2.04	88.24	98	0.49	288	698	11.86	40.66	26.06
2.12	85.4	88	0.7	292.08	588	10	42.04	22.44
2.05	92.58	101.7	0.47	241	682	12.37	42.88	20.88
2.05	92.2	102.54	0.66	333.33	544	10.88	44.66	22.68
1.88	95.44	103.52	0.65	352.32	488	10.88	38.9	24.42
2.04	88.24	98	0.49	288	698	11.86	40.66	26.06
2.12	85.4	88	0.7	292.08	588	10	42.04	22.44
2.05	92.58	101.7	0.47	241	682	12.37	42.88	20.88
2.05	92.2	102.54	0.66	333.33	544	10.88	44.66	22.68
1.88	95.44	103.52	0.65	352.32	488	10.88	38.9	24.42
2.04	88.24	98	0.49	288	698	11.86	40.66	26.06

2.12	85.4	88	0.7	292.08	588	10	42.04	22.44
2.08	154.88	138.1	1.8	390	1040	24	24	12.2
2.06	150.72	139	1.77	420	1048	20.88	24.44	12.4
2.12	147.26	142	1.75	460	1060	20.92	26	12.6
2.32	160.05	148	1.84	462	1080	20.94	26.88	12.8
2.44	158.44	147.8	1.73	482	1100	20.96	26.98	12.2
2.32	154.88	146.6	1.6	488	1040	20.98	24	12.4
2.64	150.72	142	1.44	490	1048	22	24.44	12.6
2.46	147.26	138.1	1.42	420	1060	22.24	26	12.8
2.72	160.05	139	1.54	460	1080	22.88	26.88	12.2
2.22	158.44	142	1.32	462	1100	22.98	26.98	12.4
2.16	154.88	148	1.28	482	1040	22.86	24	12.6
2.22	150.72	147.8	1.72	488	1048	22.92	24.44	12.8
2.62	147.26	146.6	1.88	490	1060	22.92	26	14.2
2.22	160.05	142	1.54	420	1080	24	26.88	14.4
2.16	158.44	138.1	1.64	460	1100	20.88	26.98	14.6
2.22	154.88	139	1.84	462	1040	20.92	24	14.6
2.62	150.72	142	1.73	482	1048	20.94	24.44	14.8
2.54	147.26	148	1.6	488	1060	20.96	26	14
2.58	160.05	147.8	1.44	490	1080	20.98	26.88	14.22
2.72	158.44	146.6	1.42	500	1100	22	26.98	14.6
2.08	154.88	138.1	1.8	390	1040	24	24	12.2
2.06	150.72	139	1.77	420	1048	20.88	24.44	12.4
2.12	147.26	142	1.75	460	1060	20.92	26	12.6
2.32	160.05	148	1.84	462	1080	20.94	26.88	12.8
2.44	158.44	147.8	1.73	482	1100	20.96	26.98	12.2
2.32	154.88	146.6	1.6	488	1040	20.98	24	12.4
2.64	150.72	142	1.44	490	1048	22	24.44	12.6
2.46	147.26	138.1	1.42	420	1060	22.24	26	12.8
2.72	160.05	139	1.54	460	1080	22.88	26.88	12.2
2.22	158.44	142	1.32	462	1100	22.98	26.98	12.4
2.16	154.88	148	1.28	482	1040	22.86	24	12.6
2.22	150.72	147.8	1.72	488	1048	22.92	24.44	12.8
2.62	147.26	146.6	1.88	490	1060	22.92	26	14.2
2.22	160.05	142	1.54	420	1080	24	26.88	14.4
2.16	158.44	138.1	1.64	460	1100	20.88	26.98	14.6
2.22	154.88	139	1.84	462	1040	20.92	24	14.6
2.62	150.72	142	1.73	482	1048	20.94	24.44	14.8
2.54	147.26	148	1.6	488	1060	20.96	26	14
2.58	160.05	147.8	1.44	490	1080	20.98	26.88	14.22
2.72	158.44	146.6	1.42	500	1100	22	26.98	14.6
2.08	154.88	138.1	1.8	390	1040	24	24	12.2
2.06	150.72	139	1.77	420	1048	20.88	24.44	12.4
2.12	147.26	142	1.75	460	1060	20.92	26	12.6
2.32	160.05	148	1.84	462	1080	20.94	26.88	12.8
2.44	158.44	147.8	1.73	482	1100	20.96	26.98	12.2
2.32	154.88	146.6	1.6	488	1040	20.98	24	12.4
2.64	150.72	142	1.44	490	1048	22	24.44	12.6
2.46	147.26	138.1	1.42	420	1060	22.24	26	12.8
2.72	160.05	139	1.54	460	1080	22.88	26.88	12.2

2.22	158.44	142	1.32	462	1100	22.98	26.98	12.4

COR	INS	CPP	HPP	SAAA	IL1	IL111	IL6	TNF
7.01	6.4	7.48	3.12	6.76	72	98	54	68
8.01	5.88	6.58	3.14	7.02	68	88	52	66
9.06	6.88	5.88	4.05	8.02	86	86	44	64
6.88	4.98	5.64	3.02	8	78	90	60	70
8.82	4.42	8.02	4.88	6	64	92	62	72
7.01	6.4	7.48	3.12	6.76	72	98	54	68
8.01	5.88	6.58	3.14	7.02	68	88	52	66
9.06	6.88	5.88	4.05	8.02	86	86	44	64
6.88	4.98	5.64	3.02	8	78	90	60	70
8.82	4.42	8.02	4.88	6	64	92	62	72
7.01	6.4	7.48	3.12	6.76	72	98	54	68
8.01	5.88	6.58	3.14	7.02	68	88	52	66
9.06	6.88	5.88	4.05	8.02	86	86	44	64
6.88	4.98	5.64	3.02	8	78	90	60	70
8.82	4.42	8.02	4.88	6	64	92	62	72
7.01	6.4	7.48	3.12	6.76	72	98	54	68
8.01	5.88	6.58	3.14	7.02	68	88	52	66
9.06	6.88	5.88	4.05	8.02	86	86	44	64
6.88	4.98	5.64	3.02	8	78	90	60	70
8.82	4.42	8.02	4.88	6	64	92	62	72
7.01	6.4	7.48	3.12	6.76	72	98	54	68
8.01	5.88	6.58	3.14	7.02	68	88	52	66
9.06	6.88	5.88	4.05	8.02	86	86	44	64
6.88	4.98	5.64	3.02	8	78	90	60	70
8.82	4.42	8.02	4.88	6	64	92	62	72
7.01	6.4	7.48	3.12	6.76	72	98	54	68
8.01	5.88	6.58	3.14	7.02	68	88	52	66
9.06	6.88	5.88	4.05	8.02	86	86	44	64
6.88	4.98	5.64	3.02	8	78	90	60	70
8.82	4.42	8.02	4.88	6	64	92	62	72
7.01	6.4	7.48	3.12	6.76	72	98	54	68
8.01	5.88	6.58	3.14	7.02	68	88	52	66
9.06	6.88	5.88	4.05	8.02	86	86	44	64
6.88	4.98	5.64	3.02	8	78	90	60	70
8.82	4.42	8.02	4.88	6	64	92	62	72
7.01	6.4	7.48	3.12	6.76	72	98	54	68
8.01	5.88	6.58	3.14	7.02	68	88	52	66
9.06	6.88	5.88	4.05	8.02	86	86	44	64
6.88	4.98	5.64	3.02	8	78	90	60	70
8.82	4.42	8.02	4.88	6	64	92	62	72
7.01	6.4	7.48	3.12	6.76	72	98	54	68
8.01	5.88	6.58	3.14	7.02	68	88	52	66
9.06	6.88	5.88	4.05	8.02	86	86	44	64
6.88	4.98	5.64	3.02	8	78	90	60	70
8.82	4.42	8.02	4.88	6	64	92	62	72
7.01	6.4	7.48	3.12	6.76	72	98	54	68
8.01	5.88	6.58	3.14	7.02	68	88	52	66
9.06	6.88	5.88	4.05	8.02	86	86	44	64
6.88	4.98	5.64	3.02	8	78	90	60	70

8.82	4.42	8.02	4.88	6	64	92	62	72
1.6	8	2	0.12	2.2	30	30	32.44	24.6
1.66	8.5	2.8	0.13	2.8	30.5	32.44	30.22	25.2
1.73	8.64	3.2	0.14	2.88	32.88	34.06	28.44	26.5
1.86	8.72	3.8	0.15	2.92	22.5	36.2	26.32	28
1.96	8.61	3.6	0.16	2.66	23.5	38.02	30.2	30
1.88	8.8	3.4	0.17	3	24.5	30	32.08	32
1.6	8.82	2.2	0.18	2.2	25.5	32.44	28.1	24.6
1.66	8.92	4.2	0.19	2.8	26.5	34.06	29.1	25.2
1.73	8.94	4.6	0.12	2.88	27.5	36.2	32.44	26.5
1.86	8.96	4.1	0.13	2.92	29.5	38.02	30.22	28
1.96	8	2	0.14	2.66	38.8	28.44	28.44	30
1.86	8.5	2.8	0.15	3	32.44	26.4	26.32	32
1.6	8.64	3.2	0.16	2.2	36.45	24.32	30.2	24.6
1.66	8.72	3.8	0.17	2.8	23.5	24.2	32.08	25.2
1.73	8.61	3.6	0.18	2.88	24.5	28.2	28.1	26.5
1.86	8.8	3.4	0.19	2.92	25.5	29.2	29.1	28
1.96	8.82	2.2	0.12	2.66	26.5	30	29.1	30
1.84	8.92	4.2	0.13	3	27.5	32.22	30	32
1.6	8.94	4.6	0.14	2.8	29.5	32.8	32	22.3
1.7	8.96	4.1	0.15	3	27.2	34.62	32.88	23.2
1.6	8	2	0.12	2.2	30	30	32.44	24.6
1.66	8.5	2.8	0.13	2.8	30.5	32.44	30.22	25.2
1.73	8.64	3.2	0.14	2.88	32.88	34.06	28.44	26.5
1.86	8.72	3.8	0.15	2.92	22.5	36.2	26.32	28
1.96	8.61	3.6	0.16	2.66	23.5	38.02	30.2	30
1.88	8.8	3.4	0.17	3	24.5	30	32.08	32
1.6	8.82	2.2	0.18	2.2	25.5	32.44	28.1	24.6
1.66	8.92	4.2	0.19	2.8	26.5	34.06	29.1	25.2
1.73	8.94	4.6	0.12	2.88	27.5	36.2	32.44	26.5
1.86	8.96	4.1	0.13	2.92	29.5	38.02	30.22	28
1.96	8	2	0.14	2.66	38.8	28.44	28.44	30
1.86	8.5	2.8	0.15	3	32.44	26.4	26.32	32
1.6	8.64	3.2	0.16	2.2	36.45	24.32	30.2	24.6
1.66	8.72	3.8	0.17	2.8	23.5	24.2	32.08	25.2
1.73	8.61	3.6	0.18	2.88	24.5	28.2	28.1	26.5
1.86	8.8	3.4	0.19	2.92	25.5	29.2	29.1	28
1.96	8.82	2.2	0.12	2.66	26.5	30	29.1	30
1.84	8.92	4.2	0.13	3	27.5	32.22	30	32
1.6	8.94	4.6	0.14	2.8	29.5	32.8	32	22.3
1.7	8.96	4.1	0.15	3	27.2	34.62	32.88	23.2
1.6	8	2	0.12	2.2	30	30	32.44	24.6
1.66	8.5	2.8	0.13	2.8	30.5	32.44	30.22	25.2
1.73	8.64	3.2	0.14	2.88	32.88	34.06	28.44	26.5
1.86	8.72	3.8	0.15	2.92	22.5	36.2	26.32	28
1.96	8.61	3.6	0.16	2.66	23.5	38.02	30.2	30
1.88	8.8	3.4	0.17	3	24.5	30	32.08	32
1.6	8.82	2.2	0.18	2.2	25.5	32.44	28.1	24.6
1.66	8.92	4.2	0.19	2.8	26.5	34.06	29.1	25.2
1.73	8.94	4.6	0.12	2.88	27.5	36.2	32.44	26.5

1.86	8.96	4.1	0.13	2.92	29.5	38.02	30.22	28

INF	IL10	MMP2	MMP9	iron	tibc	uibc	tf	feretei
7	54	40	44	86.42	348	261.58	86	24
8	62	38	48	88.02	386	297.98	84.88	22
8.6	48	38.66	38	84.5	382	297.5	80.98	20
9	68	28	38.44	88	389	301	86	18
9.2	70	32.44	45	90	380	290	90	20
8.4	54	40	44	86.42	348	261.58	86	24
6	62	38	48	88.02	386	297.98	84.88	22
7	48	38.66	38	84.5	382	297.5	80.98	20
8	68	28	38.44	88	389	301	86	18
8.6	70	32.44	45	90	380	290	90	20
9	54	40	44	86.42	348	261.58	86	24
9.2	62	38	48	88.02	386	297.98	84.88	22
8.4	48	38.66	38	84.5	382	297.5	80.98	20
7	68	28	38.44	88	389	301	86	18
8	70	32.44	45	90	380	290	90	20
8.6	54	40	44	86.42	348	261.58	86	24
9	62	38	48	88.02	386	297.98	84.88	22
9.2	48	38.66	38	84.5	382	297.5	80.98	20
8.4	68	28	38.44	88	389	301	86	18
6	70	32.44	45	90	380	290	90	20
7	54	40	44	86.42	348	261.58	86	24
8	62	38	48	88.02	386	297.98	84.88	22
8.6	48	38.66	38	84.5	382	297.5	80.98	20
9	68	28	38.44	88	389	301	86	18
9.2	70	32.44	45	90	380	290	90	20
8.4	54	40	44	86.42	348	261.58	86	24
6	62	38	48	88.02	386	297.98	84.88	22
7	48	38.66	38	84.5	382	297.5	80.98	20
8	68	28	38.44	88	389	301	86	18
8.6	70	32.44	45	90	380	290	90	20
9	54	40	44	86.42	348	261.58	86	24
9.2	62	38	48	88.02	386	297.98	84.88	22
8.4	48	38.66	38	84.5	382	297.5	80.98	20
7	68	28	38.44	88	389	301	86	18
8	70	32.44	45	90	380	290	90	20
8.6	54	40	44	86.42	348	261.58	86	24
9	62	38	48	88.02	386	297.98	84.88	22
9.2	48	38.66	38	84.5	382	297.5	80.98	20
8.4	68	28	38.44	88	389	301	86	18
6	70	32.44	45	90	380	290	90	20
7	54	40	44	86.42	348	261.58	86	24
8	62	38	48	88.02	386	297.98	84.88	22
8.6	48	38.66	38	84.5	382	297.5	80.98	20
9	68	28	38.44	88	389	301	86	18
9.2	70	32.44	45	90	380	290	90	20
8.4	54	40	44	86.42	348	261.58	86	24
6	62	38	48	88.02	386	297.98	84.88	22
7	48	38.66	38	84.5	382	297.5	80.98	20
8	68	28	38.44	88	389	301	86	18

8.6	70	32.44	45	90	380	290	90	20
2	100	14.5	21.3	102.42	256.06	153.64	124	16
2.4	98	15.5	22.3	107.27	262.33	155.06	125	15
1.8	102	16.3	23.3	106.44	279.3	172.86	126	17
2.88	106	14.5	24.1	103.44	282.06	178.62	127	18
3	110	15.5	21.3	102.42	242.15	139.73	128	16
3.2	99.8	16.3	22.3	107.27	226.46	119.19	129	15
2	100	14.5	23.3	106.44	298.65	192.21	121	17
2.4	98	15.5	24.1	103.44	244.84	141.4	128	18
1.8	102	16.3	21.3	102.42	256.06	153.64	129	14
2.88	106	14.5	22.3	107.27	262.33	155.06	124	16
3	110	15.5	23.3	106.44	279.3	172.86	125	16
3.2	99.8	16.3	24.1	103.44	282.06	178.62	126	15
2	100	14.5	21.3	102.42	242.15	139.73	127	14
2.4	98	15.5	22.3	107.27	226.46	119.19	128	14
1.8	102	16.3	23.3	106.44	298.65	192.21	129	16
2.88	106	14.5	24.1	103.44	244.84	141.4	121	15
3	110	15.5	21.3	102.42	256.06	153.64	128	17
3.2	99.8	16.3	22.3	107.27	262.33	155.06	129	18
2.6	101.3	14.5	23.3	106.44	279.3	172.86	130	14
2.64	101.2	15.5	24.1	103.44	282.06	178.62	122	16
2	100	14.5	21.3	102.42	256.06	153.64	124	16
2.4	98	15.5	22.3	107.27	262.33	155.06	125	15
1.8	102	16.3	23.3	106.44	279.3	172.86	126	17
2.88	106	14.5	24.1	103.44	282.06	178.62	127	18
3	110	15.5	21.3	102.42	242.15	139.73	128	16
3.2	99.8	16.3	22.3	107.27	226.46	119.19	129	15
2	100	14.5	23.3	106.44	298.65	192.21	121	17
2.4	98	15.5	24.1	103.44	244.84	141.4	128	18
1.8	102	16.3	21.3	102.42	256.06	153.64	129	14
2.88	106	14.5	22.3	107.27	262.33	155.06	124	16
3	110	15.5	23.3	106.44	279.3	172.86	125	16
3.2	99.8	16.3	24.1	103.44	282.06	178.62	126	15
2	100	14.5	21.3	102.42	242.15	139.73	127	14
2.4	98	15.5	22.3	107.27	226.46	119.19	128	14
1.8	102	16.3	23.3	106.44	298.65	192.21	129	16
2.88	106	14.5	24.1	103.44	244.84	141.4	121	15
3	110	15.5	21.3	102.42	256.06	153.64	128	17
3.2	99.8	16.3	22.3	107.27	262.33	155.06	129	18
2.6	101.3	14.5	23.3	106.44	279.3	172.86	130	14
2.64	101.2	15.5	24.1	103.44	282.06	178.62	122	16
2	100	14.5	21.3	102.42	256.06	153.64	124	16
2.4	98	15.5	22.3	107.27	262.33	155.06	125	15
1.8	102	16.3	23.3	106.44	279.3	172.86	126	17
2.88	106	14.5	24.1	103.44	282.06	178.62	127	18
3	110	15.5	21.3	102.42	242.15	139.73	128	16
3.2	99.8	16.3	22.3	107.27	226.46	119.19	129	15
2	100	14.5	23.3	106.44	298.65	192.21	121	17
2.4	98	15.5	24.1	103.44	244.84	141.4	128	18
1.8	102	16.3	21.3	102.42	256.06	153.64	129	14

2.88	106	14.5	22.3	107.27	262.33	155.06	124	16

tfper
24.83
22.8
22.12
22.62
23.68
24.83
22.8
22.12
22.62
23.68
24.83
22.8
22.12
22.62
23.68
24.83
22.8
22.12
22.62
23.68
24.83
22.8
22.12
22.62
23.68
24.83
22.8
22.12
22.62
23.68
24.83
22.8
22.12
22.62
23.68
24.83
22.8
22.12
22.62
23.68
24.83
22.8
22.12
22.62
23.68
24.83
22.8
22.12
22.62

23.68
40
40.89
38.11
36.67
42.3
47.37
35.64
42.25
40
40.89
38.11
36.67
42.3
47.37
35.64
42.25
40
40.89
38.11
36.67
40
40.89
38.11
36.67
42.3
47.37
35.64
42.25
40
40.89
38.11
36.67
42.3
47.37
35.64
42.25
40
40.89
38.11
36.67
40
40.89
38.11
36.67
42.3
47.37
35.64
42.25
40

40.89
