# Supplementary figures and images for: Sheep Pox Susceptibility: Role of Genetic Variants, Gene Expression, and Immune-Oxidative Markers
Source: Vet Sci. 2025 Sep 8;12(9):867. doi: 10.3390/vetsci12090867 (PMC12474403; doi:10.3390/vetsci12090867)

**Fig. Roc curve of of the estimated cytokines, APPs, MMPs, Cu, Zn and TAC in DG compared to CG.**

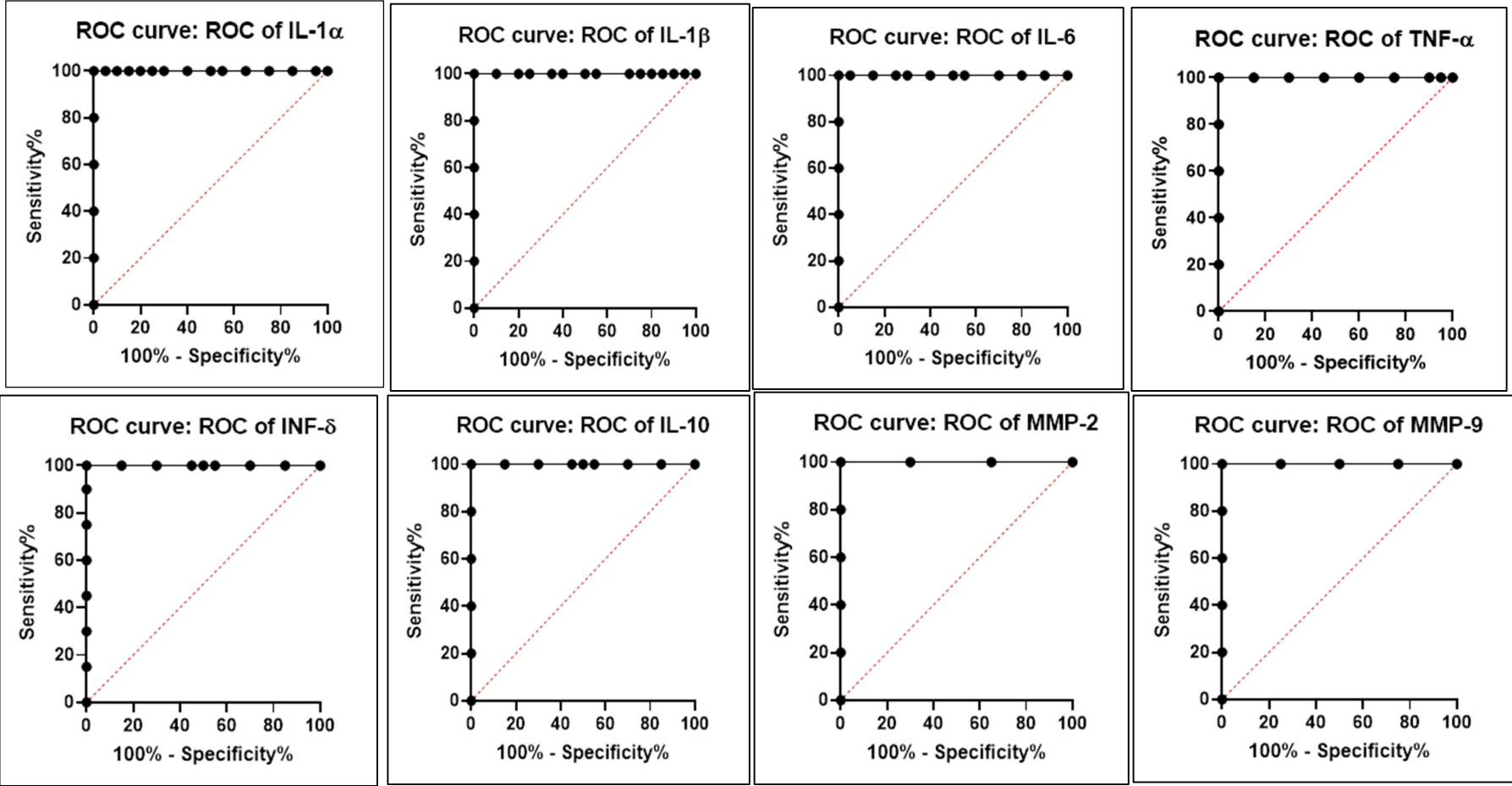

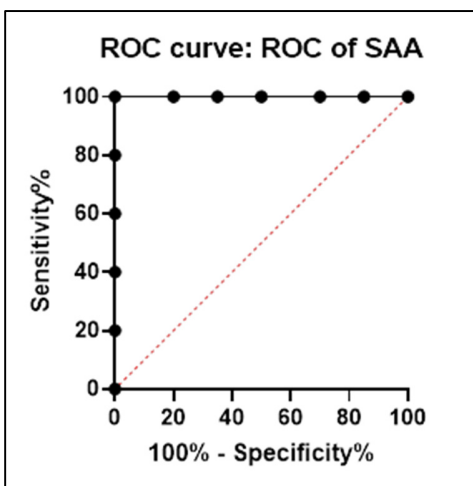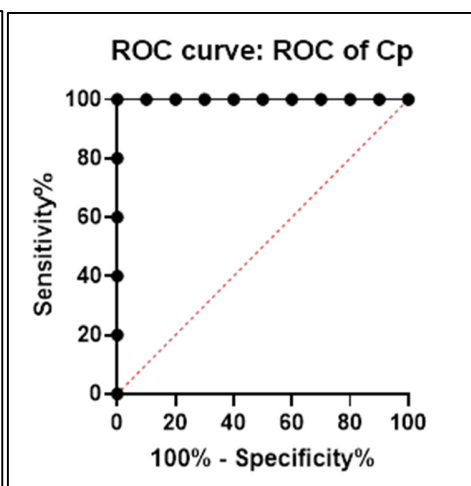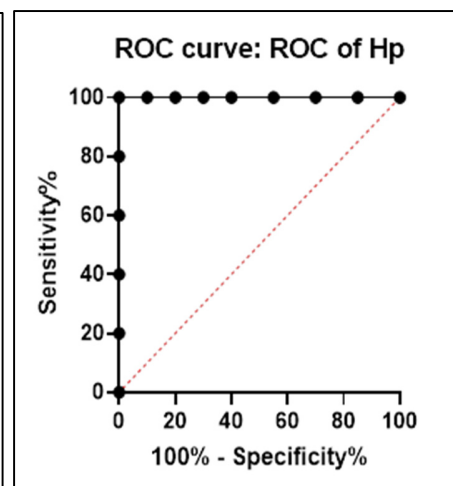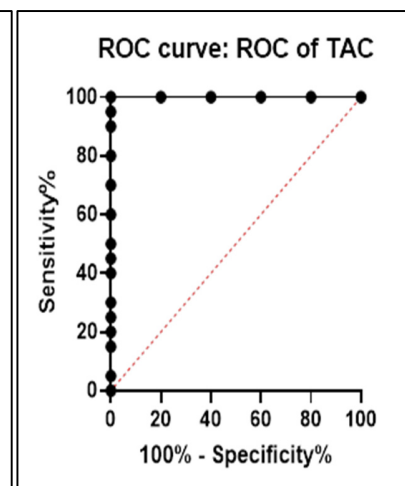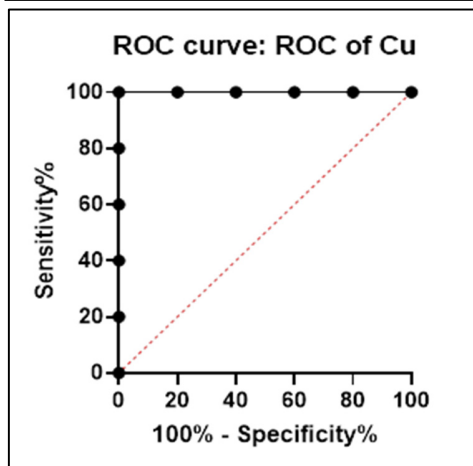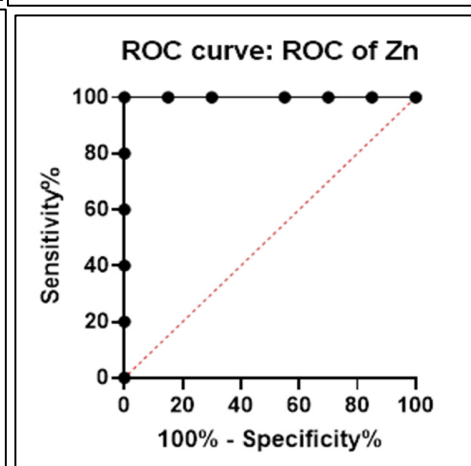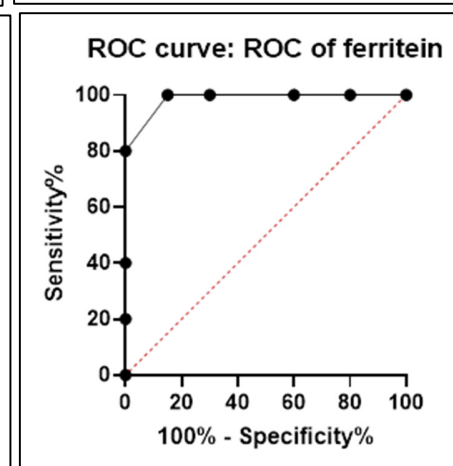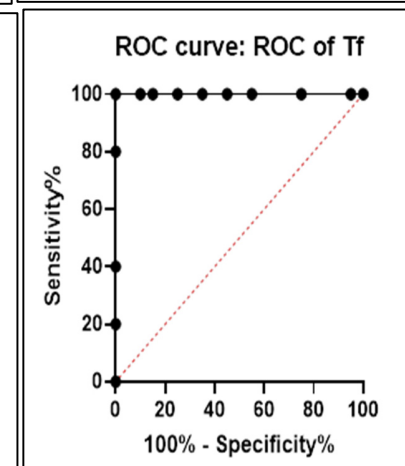

Supplement: Supplementary file 1 [file vetsci-12-00867-s001.zip › vetsci-3839312-Supplementry Figure S1, S2).pdf]
